# Hybrid cell line development system utilizing site-specific integration and methotrexate-mediated gene amplification in Chinese hamster ovary cells

**DOI:** 10.3389/fbioe.2022.977193

**Published:** 2022-09-15

**Authors:** Honggi Min, Seul Mi Kim, Dongwoo Kim, Solhwi Lee, Sumin Lee, Jae Seong Lee

**Affiliations:** ^1^ Department of Molecular Science and Technology, Ajou University, Suwon, South Korea; ^2^ Department of Applied Chemistry and Biological Engineering, Ajou University, Suwon, South Korea

**Keywords:** Chinese hamster ovary, CRISPR/Cas9, DHFR knockout, Gene Amplification, methotrexate, targeted integration

## Abstract

Site-specific integration has emerged as a promising strategy for streamlined and predictable Chinese hamster ovary (CHO) cell line development (CLD). However, the low specific productivity of the targeted integrants limits their practical application. In this study, we developed a hybrid CLD platform combining site-specific integration of a transgene and dihydrofolate reductase/methotrexate (DHFR/MTX)-mediated gene amplification to generate high-producing recombinant CHO cell lines. We used the CRISPR/Cas9-based recombinase-mediated cassette exchange landing pad platform to integrate the *DHFR* expression cassette and transgene landing pad into a CHO genomic hot spot, *C12orf35* locus, of *DHFR*-knockout CHO-K1 host cell lines. When subjected to various MTX concentrations up to 1 μM, EGFP-expressing targeted integrants showed a 3.6-fold increase in EGFP expression in the presence of 200 nM MTX, accompanied by an increase in the *DHFR* and *EGFP* copy number. A single-step 200 nM MTX amplification increased the specific monoclonal antibody (mAb) productivity (*q*_*mAb*_) of recombinant mAb-producing targeted integrants by 2.8-folds, reaching a *q*_*mAb*_ of 9.1–11.0 pg/cell/day. Fluorescence *in situ* hybridization analysis showed colocalization of *DHFR* and mAb sequences at the intended chromosomal locations without clear amplified arrays of signals. Most MTX-amplified targeted integrants sustained recombinant mAb production during long-term culture in the absence of MTX, supporting stable gene expression in the amplified cell lines. Our study provides a new CLD platform that increases the productivity of targeted integrants by amplifying the transgene copies.

## Introduction

Chinese hamster ovary (CHO) cells represent the mammalian cell type of choice for producing recombinant therapeutic proteins, such as recombinant monoclonal antibodies (mAbs). Stable recombinant CHO (rCHO) cell line development (CLD) is crucial for large-scale manufacture of recombinant therapeutic proteins. The generation of stable pools or clonal cell lines with random integration of transgenes coupled with proper selection systems is a routine CLD process that has been used for decades (Shin and Lee, 2020). Dihydrofolate reductase (DHFR)-mediated gene amplification and glutamine synthetase (GS)-mediated gene selection systems are frequently used in the biopharmaceutical industry for high-level, stable production of recombinant therapeutic proteins (Cacciatore et al., 2010; Noh et al., 2013).

The DHFR-mediated gene amplification system is based on the DHFR enzyme, which is a metabolic catalyst for the conversion of folic acid into tetrahydrofolate. The absence of DHFR activity renders cells incapable of *de novo* synthesis of thymidine and hypoxanthine, thus, limiting the growth and proliferation of cells and eventually leading to cell death, without the addition of nucleotide precursors (Shin and Lee, 2020). This trait has been successfully adapted to rCHO CLDs by utilizing DHFR-deficient auxotrophic host cells, including CHO-DXB11 and CHO-DG44 (Wurm, 2013). The introduction of a functional *DHFR* gene together with the target gene of interest (GOI) via transfection of an expression vector allows for the selection of the transfected population and the subsequent stable cell populations in media deficient in glycine, hypoxanthine, and thymidine (Urlaub and Chasin, 1980). Along with the selection, the addition of a DHFR inhibitor, methotrexate (MTX), not only improves the selection stringency but also induces amplification of *DHFR* to develop resistance to MTX (Kaufman and Schimke, 1981). The GOI can be coamplified with *DHFR*, resulting in an increase in transgene copy number and productivity (Kim et al., 1998; Jiang et al., 2006; Chusainow et al., 2009; Cacciatore et al., 2010).

Although this traditional CLD method has been widely used, uncontrolled integration sites and transgene rearrangements in random integrants lead to high levels of heterogeneity among clonal cell lines, referred to as clonal variation, which necessitates extensive clone screening efforts (Lee et al., 2019). Moreover, MTX-mediated gene-amplified random cell lines occasionally show extensive chromosomal rearrangements and production instability, accompanied by a decrease in transgene copy number, particularly in the absence of selection pressure (Kim et al., 1998; Chusainow et al., 2009; Baik and Lee, 2017).

To mitigate the limitations of the traditional CLD method, site-specific integration-based CLD using genome editing technology, including clustered regularly interspaced short palindromic repeats (CRISPR)/CRISPR-associated protein 9 (Cas9), has recently been adopted as an alternative method (Shin and Lee, 2020). The integration of transgenes into designated genomic sites can reduce clonal variation in transgene expression, and homology-directed targeted integration (TI) enables controlled integration of intact transgene sequences without unwanted plasmid backbone DNA (Lee et al., 2015; Lee et al., 2019). Furthermore, the combination of CRISPR/Cas9-mediated TI with recombinase-mediated cassette exchange (RMCE) enables the generation of isogenic and retargetable master cell lines containing landing pads equipped with reporter/selection markers and recombinase recognition sites (Grav et al., 2018; Pristovšek et al., 2019). Once the master cell lines undergo RMCE with the RMCE donor cassette harboring the GOIs and the corresponding recombinase, recombinant cell lines producing various target proteins can be established rapidly, in which GOIs are expressed from identical and predetermined loci. However, targeted integrants often exhibit low expression levels of transgenes derived from a limited number of transgene copies. To overcome this limitation, genomic hot spots, which ensure high and stable transcriptional capability, have been sought using experimental approaches based on randomly generated high producers (Hamaker and Lee, 2018; Pristovšek et al., 2019; Dhiman et al., 2020). In addition to screening hot spots, multicopy TI methods based on the TI/RMCE hybrid system have been developed to increase transgene copy number by integrating of multiple copies of the transgene expression cassette into a single genomic site as well as multiple genomic sites (Gaidukov et al., 2018; Sergeeva et al., 2020). In a dual-RMCE master cell line, four copies of the GOI were introduced site-specifically, achieving a specific productivity (*q*_*p*_) of 12–14 pg/cell/day (pcd) and a titer of approximately 1 g/L (Sergeeva et al., 2020). Despite the potential of the current TI-mediated CLD platforms for efficient protein production, a number of constraints still exist: 1) a limited number of publicly available genuine hot spots (Hamaker and Lee, 2018); 2) context-dependent effect of vector regulatory elements at certain hot spots (Lee et al., 2018; Pristovšek et al., 2019; Sergeeva et al., 2020), requiring optimization and evaluation of each element in each target site; and 3) technical issues for multicopy TI, including low integration efficiency and the challenge of verifying the insertion of large multicopy constructs (Sergeeva et al., 2020).

In this study, we developed a new CLD system that combined the CRISPR/Cas9-based RMCE landing pad platform and the DHFR/MTX gene amplification system. To implement MTX-mediated gene amplification at a defined locus, we generated *DHFR*-knockout (KO) CHO-K1 host cell lines using CRISPR/Cas9. Then, the *DHFR* expression cassette and an RMCE landing pad harboring an *EGFP* reporter gene expression cassette flanked by the lox recombination sites were integrated into the prevalidated CHO genomic hot spot, *C12orf35* locus (Zhao et al., 2018). Based on *DHFR* ^−^ RMCE landing pad master cell lines, we evaluated the feasibility of DHFR-mediated transgene amplification at a specific locus. Furthermore, we characterized recombinant mAb-producing targeted integrants during the DHFR-mediated gene amplification process, including specific mAb productivity (*q*_*mAb*_), relative gene copy numbers, mRNA levels, chromosomal locations of transgenes, and long-term production stability.

## Materials and methods

### Plasmid construction

All the plasmids used in this study are listed in Supplementary Table S1. sgRNAs were designed using CRISPOR bioinformatics tool (version 4.95; Concordet and Haeussler, 2018) and cloned into pU6-(BbsⅠ) CBh-Cas9-T2A-mCherry (Addgene plasmid #64324). Two complementary oligonucleotides (forward: CACCG-N_20_, reverse: AAAC-N_20_-C) were annealed and ligated onto the digested #64324 using T4 ligase (Thermo Fisher Scientific, Waltham, MA), resulting in the sgRNA-Cas9 plasmid. RMCE donor and homology-directed repair (HDR) donor plasmids were constructed using uracil-specific excision reagent (USER) cloning. All primers used for USER cloning are listed in Supplementary Table S2. Five plasmids, pcDNA3.1-CMVcore-chDHFR, pcDNA3.1-LoxP-CMV-EGFP-Lox2272, pcDNA3.4-CMV-Dupilumab_HC, pcDNA3.4-CMV-Dupilumab_LC (Kim et al., 2019), and pJ204-loxP-100RPU.2-ETN-BGHpA-cHS4-100RPU.2-ETN-lox2272 (Addgene plasmid #154834; Sergeeva et al., 2020) were used as PCR templates for the RMCE donors, pcDNA3.1-CMVcore-chDHFR-LoxP-CMV-EGFP-Lox2272 and pcDNA3.1-LoxP-CMV-Dupilumab_HC-BGHpA-cHS4-CMV-Dupilumab_LC-Lox2272. The *chDHFR* and homologous region of *C12orf35* were amplified from CHO-K1 cDNA and genomic DNA, respectively, using Phusion Hot Stat Ⅱ DNA polymerase (Thermo Fisher Scientific) and were used to produce the DNA bricks for USER cloning. The BGHpA and plasmid backbone were amplified from pcDNA3.1 (Thermo Fisher Scientific), and the CMV core, LoxP-CMV-EGFP-Lox2272, and SV40pA were amplified from pEGFP-C1 (Clontech, Palo Alto, CA). Dupilumab_HC or Dupilumab_LC was amplified from pcDNA3.4-CMV-Dupilumab_HC or pcDNA3.4-CMV-Dupilumab_LC, respectively. The HS4 insulator was amplified from #154834. The NLS-Cre-NLS vector has been described in a previous study (Shin et al., 2021). An RMCE donor integrating the *C12orf35* locus was constructed by combining CMVcore-DHFR-BGHpA and LoxP-CMV-EGFP-Lox2272-SV40pA. PCR amplicons were purified using the NucleoSpin® Gel and PCR Cleanup Kit (Macherey-Nagel, Duren, Germany) and assembled with the pcDNA3.1 backbone using the USER enzyme (New England Biolabs, Ipswich, MA). The assembled DNA bricks were transformed into *Escherichia coli* One Shot® Mach1™ competent cells (Thermo Fisher Scientific). All plasmids were verified by Sanger sequencing and purified using NucleoBond Xtra Midi EF (Macherey-Nagel), according to the manufacturer’s instructions.

### Cell lines and culture maintenance

Adherent CHO-K1 cell lines were maintained in Dulbecco’s modified Eagle’s medium (DMEM, Gibco, Gaithersburg, MD) supplemented with 10% (v/v) fetal bovine serum (FBS, Hyclone, Logan, UT). Recombinant CHO-K1 cell lines expressing DHFR, EGFP, or dupilumab were maintained in DMEM supplemented with 10% dialyzed FBS (dFBS, Thermo Fisher Scientific). The cells were maintained in monolayer cultures in 25 cm^2^ T-flasks (Nunc, Roskilde, Denmark) with a working volume of 5 ml and incubated at 37°C in a humidified 5% CO_2_ atmosphere. The cell lines that were amplified by MTX were maintained in the maintenance media supplemented with 20, 50, 100, 200 nM, or 1 μM MTX (Sigma-Aldrich, St. Louis, MO). Viable cell density and viability were measured with the trypan blue dye exclusion method using an automated cell counter (Countess II FL; Invitrogen, Carlsbad, CA).

### Generation and validation of *DHFR* ^−^ CHO-K1 cell line

The NEPA21 electroporator (Nepagene, Chiba, Japan) was used to transfect 1.0×10^6^ cells of CHO-K1 with 10 µg of sgDHFR-Cas9 plasmid as described previously (Kyeong and Lee, 2022). After 3 days, the limiting dilution method was performed in 96-well culture plates. Every single clone generated in the 96-well plates was split into two 96-well plates containing DMEM supplemented with 10% dFBS with or without 1× hypoxanthine-thymidine (HT; Thermo Fisher Scientific) to select for *DHFR* ^−^ CHO-K1 clones via HT-dependency screening (Lee et al., 2021). To confirm the modifications of the genomic *DHFR* sequence, genomic DNA was extracted from the HT-auxotrophic clones using QuickExtract™ (Lucigen, Teddington, United Kingdom) and amplified using primers specific to the *DHFR* target region. The amplicons were purified and sequenced by Sanger sequencing. The PCR primers used are listed in Supplementary Table S2.

### Generation of landing pad master cell line

The *DHFR* ^−^ CHO-K1 cell line was cotransfected with 5 µg of sgC12orf35-Cas9 vector targeting the *C12orf35* locus and 5 µg of HDR donor plasmid, including two expression cassettes that expressed *chDHFR* and *EGFP*, using the NEPA21 electroporator. The transfected cell pool was maintained in a 6-well culture plate without HT supplement for 2 weeks to generate stable cell pools, followed by limiting dilution. EGFP^+^ monoclonal cell lines were selected using a ZOE fluorescent cell imager (Bio-Rad Laboratories, Hercules, CA). Subsequently, the genomic DNA of the selected clones was extracted using QuickExtract™, and 5ʹ/3ʹ-junction PCR was performed to verify knock-in (KI) using primers–targeting exon 1 of *Kiaa1551* and *chDHFR* for 5′ junction and *EGFP* and exon 2 of *Kiaa1551* for 3′ junction–and Phusion High-Fidelity PCR Master Mix (Thermo Fisher Scientific) by touchdown PCR method (98°C for 30 s; 10×: 98°C for 10 s, 68–58°C [−1°C/cycle] for 30 s, 72°C for 40 s; 30×: 98°C for 10 s, 58°C for 30 s, 72°C for 40 s; 72°C for 10 min). The PCR primers used are listed in Supplementary Table S2.

### Generation of recombinant mAb-producing rCHO cell lines

The landing pad master cell line was cotransfected with 7.5 μg mAb RMCE donor plasmid and 2.5 μg NLS-Cre-NLS vector using the NEPA21 electroporator. After 13 days, mAb-producing cell pools were sorted using a FACS sorter (BD FACSAria™ III Cell Sorter; Becton Dickinson, Franklin Lakes, NJ) to isolate the EGFP^–^ population. EGFP^–^ monoclonal cell lines were isolated using limiting dilution of the cell pools. Clones were expanded and verified by 5′/3′-junction PCR as previously described.

### MTX amplification

DHFR-mediated gene amplification via MTX selection was performed in the landing pad master cell line and the recombinant mAb-expressing rCHO cell line. Cells were seeded at a concentration of 2×10^5^ cells/mL in 6-well culture plates containing 3 ml of selection medium with the corresponding concentration of MTX. MTX selection was terminated when the wells of the culture plate were full.

### Flow cytometry

Landing pad master cell lines and their MTX-amplified pool cells were subcultured every 3 days at a seeding concentration of 2×10^5^ cells/mL for three passages. The remaining cells were resuspended in phosphate-buffered saline supplemented with 10% FBS and analyzed on a FACSCalibur (Becton Dickinson) or NovoCyte Flow Cytometer (Agilent Technologies, Santa Clara, CA), equipped with a 530/30 detection filter and a blue laser emitting at 488 nm. Cells were gated by forward scatter versus side scatter plots, and the fluorescence threshold was set based on a CHO-K1 (EGFP-negative control) at approximately 0.1%. Flow cytometry data were analyzed using FlowJo (Tree Star, OR) and NovoExpress software (Agilent Technologies).

### Quantitative real-time PCR

Landing pad master cell lines and 200 nM MTX-amplified pool cells for each clone were harvested on day 3 after the medium exchange. The recombinant mAb-producing rCHO cell lines and 200 nM MTX-amplified pools for each clone were harvested on day 4 during batch culture. The genomic DNA from cell pellets was purified using the GeneJET Genomic DNA Purification Kit (Thermo Fisher Scientific). mRNA was extracted using the RNeasy Mini Kit (Qiagen, Hilden, Germany), followed by cDNA synthesis using the Maxima First Strand cDNA Synthesis kit (Thermo Fisher Scientific). The relative gene copy number and mRNA expression levels were analyzed using the CFX96 Real-Time System (Bio-Rad) or StepOnePlus Real-Time PCR System (Applied Biosystems, Waltham, MA), and Power SYBR Green Master Mix (Applied Biosystems). Amplification was performed with the following conditions: 95°C for 10 min, 40× 95°C for 15 s, and 60°C for 1 min. The primer sequences used are listed in Supplementary Table S2. The relative values were calculated using the ΔΔCT method, which was normalized to *vinculin*.

### Western blot analysis

One to two million cells were pelleted and lysed using 1× RIPA lysis buffer (Rockland Immunochemicals, Inc., Limerick, PA) containing 1× protease inhibitor cocktail kit5 (Bio-Rad). The total protein concentration of cell lysates was determined using the Pierce BCA Protein Assay Kit (Thermo Fisher Scientific), and 11.5 μg of protein was reduced using Bolt™ Sample Reducing Agent (Invitrogen), according to the manufacturer’s instructions. The reduced proteins were loaded into Bolt™ 4–12% Bis-Tris Plus Gels (Thermo Fisher Scientific). The gel was run in MES buffer at 175 V for 35 min and then transferred to a membrane using an iBlot2 Dry Blotting System (Thermo Fisher Scientific). The transferred membranes were blocked with a 5% skim milk solution (Becton Dickinson) for 1 h at 15 rpm. Primary antibody incubation was performed overnight at 4°C at 11 rpm using β-actin rabbit mAb (4970T, Cell Signaling Technology, MA) and mouse anti-DHFR antibody (sc-377091, Santa Cruz Biotechnology, Dallas, TX) at a 1:1000 dilution. Primary antibodies were washed with cold TBS-T buffer, and secondary antibody incubation was carried out at 30 rpm for 10 min using HRP-linked anti-rabbit IgG (7074S, Cell Signaling Technology) and anti-mouse IgG (7076S, Cell Signaling Technology) at a 1:2000 dilution. Protein bands were detected using ECL western blotting detection reagents (GE Healthcare, Chicago, IL) and ChemiDoc (Bio-Rad).

### Fluorescence in situ hybridization analysis

Cells were treated with colcemid (0.02 μg/ml) for 20 h and harvested for chromosome preparation. After treatment with 0.075 M KCl for 20 min at 37°C, the cells were fixed three times with MeOH:acetic acid (3:1), and the fixed cells were subsequently spread on slides. FISH probes were generated by direct labelling of pcDNA3.1-LoxP-CMV-Dupilumab_HC-BGHpA-cHS4-CMV-Dupilumab_LC-Lox2272 and pcDNA3.1-CMVcore-chDHFR with Green-dUTP and Cy3-dUTP, respectively, through nick translation. The labelled probes were mixed with sonicated salmon sperm DNA in hybridization solution. For FISH analysis, the probe was applied to cell spreads, denatured at 70°C for 5 min, and hybridized overnight at 37°C. The hybridized slides were washed and counterstained with DAPI. After mounting with an antifade mountant, FISH images were captured using the CW4000 FISH application program (Leica Microsystems Imaging Solution Ltd., Cambridge, United Kingdom) using a cooled CCD camera mounted on a Leica DMRA2 microscope.

### Batch culture

Recombinant mAb-producing rCHO cell lines and their corresponding 200 nM MTX-amplified pool cells were seeded at a concentration of 3×10^5^ cells/mL in 6-well culture plates. Cultures were independently performed three times. On day 4, the wells were sacrificed for the measurement of viable cell concentration using the trypan blue dye exclusion method. Culture supernatants were sampled and stored at −70°C until further analysis. The recombinant mAb concentrations of the clones and their 200 nM MTX selection pool cells were measured using an enzyme-linked immunosorbent assay, as previously described (Kyeong and Lee, 2022). Briefly, samples were loaded onto microtiter plates (Thermo Fisher Scientific) coated with anti-human IgG (Sigma-Aldrich). Peroxidase-labelled goat anti-human IgG (Fc specific, Sigma-Aldrich) was used as an enzyme antibody conjugate. Next, 3,3′,5,5′ -tetramethylbenzidine (TMB, Sigma-Aldrich) was added to wells as a chromogenic substrate for peroxidase. The reaction was stopped by adding H_2_SO_4_. The plates were washed three times with phosphate buffered saline containing 0.05% Tween20 at every step. The OD value at 450 nm was measured using a Synergy HTX Multi-Mode Microplate Reader (BioTek, Winooski, VT). The *q*_*mAb*_ was calculated based on the integral viable cell concentration and mAb concentration on day 4, as previously described (Kyeong and Lee, 2022). *q*_*mAb*_ was calculated as the slope of the linear regression line through data points of integral viable cell concentration (*x*-axis) and mAb concentration (*y*-axis) measurements.

### Long-term culture

MTX-amplified pool cells of recombinant mAb-producing rCHO cell lines were cultured at a seeding concentration of 2×10^5^ cells/mL in 6-well culture plates containing 3 ml selection medium in the presence or absence of 200 nM MTX. Cells were subcultured every 4 days for 15 passages for approximately 2 months. Culture supernatants were harvested and stored at −70°C for determination of the mAb concentration.

### Statistical analysis

The results are expressed as mean ± standard deviation. Unpaired two-tailed *t*-tests and two-way analysis of variance followed by Dunnett’s multiple comparisons were performed using GraphPad Prism 8 software (GraphPad Software, Inc., San Diego, CA) to determine the significance of the results. Differences between means were considered statistically significant at *p* < 0.05.

## Results

### Generation of *DHFR* ^−^ CHO-K1 cell line

**FIGURE 1:**
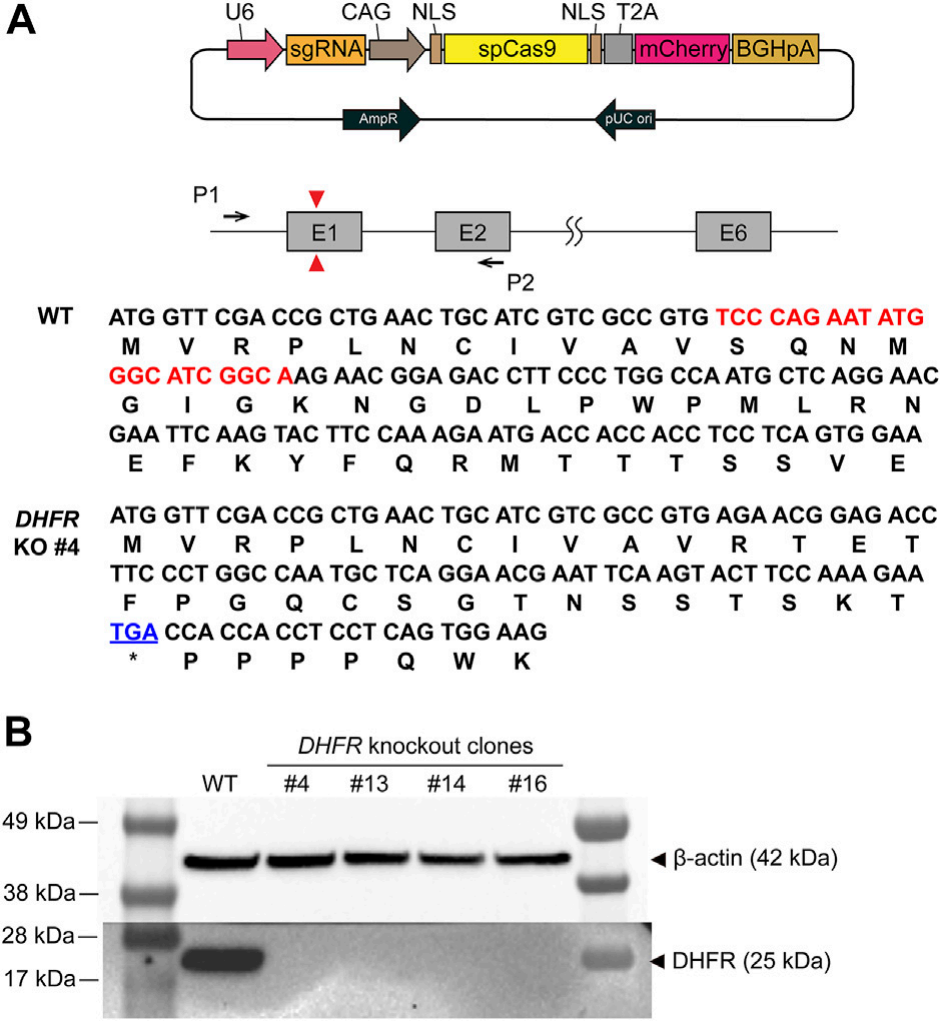
Generation and validation of the dihydrofolate reductase (*DHFR*) knockout (KO) clones. **(A)** Schematic illustration of *DHFR* KO in Chinese hamster ovary (CHO)-K1 wild type (WT). Red triangles represent the position of the sgRNA target site and black arrows denote the set of PCR primers used to confirm *DHFR* sequence. The DNA and amino acid sequences indicate the coding region of *DHFR* from WT and *DHFR* ^−^ clone 4. The deleted region of *DHFR* in the genome of clone 4 is marked in red. The premature stop codon is shown in blue. **(B)** Western blot analysis of DHFR in CHO-K1 WT, *DHFR* ^−^ clone 4, 13, 14, and 16. β-actin was used as a loading control.

To implement the DHFR/MTX gene amplification system in CHO-K1, we first knocked out endogenous *DHFR* in CHO-K1 cells using the CRISPR/Cas9 system. A guide RNA was designed to generate insertion/deletion mutations in the first exon, leading to frameshift mutations and functional gene KO (Figure 1A). CHO-K1 cells were transfected with sgDHFR-Cas9 vectors, followed by limiting dilution to obtain clonal cells. We then split and cultured the clones in a medium supplemented with 10% dFBS with or without HT supplement for 10 days. Four of the 16 clones showed no growth in the absence of HT because HT is an essential substrate for the salvage pathway of nucleotide biosynthesis. Western blot analysis of the four clones confirmed the absence of DHFR expression (Figure 1B). To further validate the disruption of the genomic *DHFR* sequence in the four clones, PCR products of *DHFR* from these clones were sequenced. Among the four clones, sequencing results indicated that one clone (*DHFR* ^−^ CHO-K1 #4) harbored a 22-bp deletion in all copies of *DHFR* exon 1, which induced a frameshift mutation and premature stop codon (Figure 1A), whereas the other three clones had multiple patterns (Supplementary Table S3). Therefore, *DHFR* ^−^ CHO-K1 #4 was selected as the host cell line for DHFR/MTX gene amplification in the DHFR-KI hybrid system.

### Site-specific integration of *DHFR* expression cassette and RMCE landing pad

**FIGURE 2:**
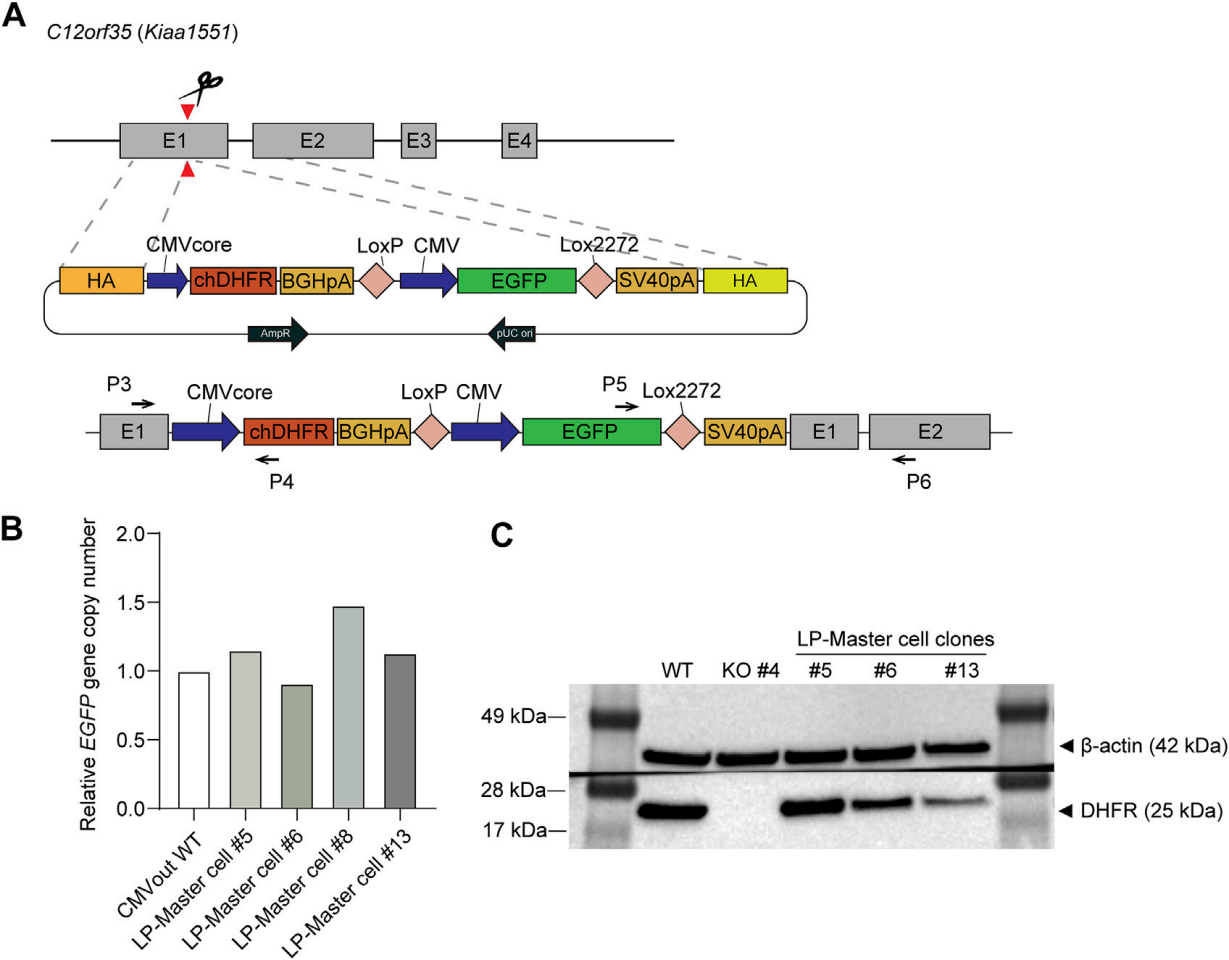
Generation and validation of the dihydrofolate reductase (*DHFR*)^−^ recombinase-mediated cassette exchange (RMCE) landing pad master cell line. **(A)** Schematic illustration of targeted integration of *DHFR* expression cassette and RMCE landing pad (LP) at *C12orf35* locus. The Chinese hamster *DHFR* is expressed at a low level of expression by CMV core promoter, and EGFP is expressed by CMV promoter. LoxP and Lox2722 recombination sites flanked CMV-EGFP, providing poly A trapping for RMCE. Primer positions for 5'/3′-junction PCR were denoted as black arrows. **(B)** Genotyping qPCR of *EGFP* gene copy number in *DHFR* ^−^ RMCE landing pad master cell line. It was normalized to a reference cell line, CMVout WT, which contained a single-copy *EGFP* in the genome. **(C)** Western blot analysis of DHFR in the cell lysates of *DHFR* ^−^ clone 4 (WT) and landing pad master cell lines. β-actin was used as a loading control.

When located near the *DHFR* gene region, a GOI can be amplified using the DHFR/MTX gene amplification system. To generate *DHFR* and GOI expression cassettes in the same chromosome location, we performed HDR-mediated TI using the CRISPR/Cas9 system. *DHFR* ^−^ CHO-K1 #4 clone was transfected with sgC12orf35-Cas9 vector and HDR donor plasmid harboring *DHFR* and *EGFP* expression cassettes (Figure 2A). sgRNA of the *C12orf35* locus targets exon 1 of the Chinese hamster *Kiaa1551* gene and allows homology-mediated TI with Cas9 proteins and HDR donors. *DHFR* is expressed by a CMV core promoter with a relatively low expression to maximize DHFR-mediated gene amplification. To generate the RMCE landing pad, LoxP and Lox2272 sequences were located on both sides of the *EGFP* gene. They were recognized by Cre recombinases and mediate RMCE in the genome.

Next, to determine which cells expressed EGFP and DHFR proteins, we performed pool selection and limiting dilution to obtain TI clones. The transfected cell pool was maintained in a medium supplemented with 10% dFBS and HT during electroporation. After one subculture, the medium was changed to HT-deficient medium, followed by 6 days of selection and limiting dilution. Based on EGFP expression from the KI donor, we selected four clones that were both EGFP^+^ and 5′/3′-junction PCR-positive (Supplementary Figure S1). For an accurate comparison of gene amplification, we required single-copy TI clones prior to MTX selection; therefore, the relative copy number of *EGFP* was measured using qRT-PCR (Figure 2B). It was then normalized to the reference cell line, which contained a single-copy *EGFP* in the genome (Lee et al., 2018). Following qRT-PCR analysis, three clones, #5, #6, and #13, were selected as master cells containing the RMCE landing pad. These clones were further validated by western blot analysis for EGFP and DHFR expression (Figure 2C). EGFP expression levels were sustained during long-term cultures for approximately 2 months in the absence of selection pressure with the use of FBS, confirming stable gene expression at the *C12orf35* locus (Supplementary Figure S2).

### Characterization of DHFR-mediated gene amplification at a defined locus

**FIGURE 3:**
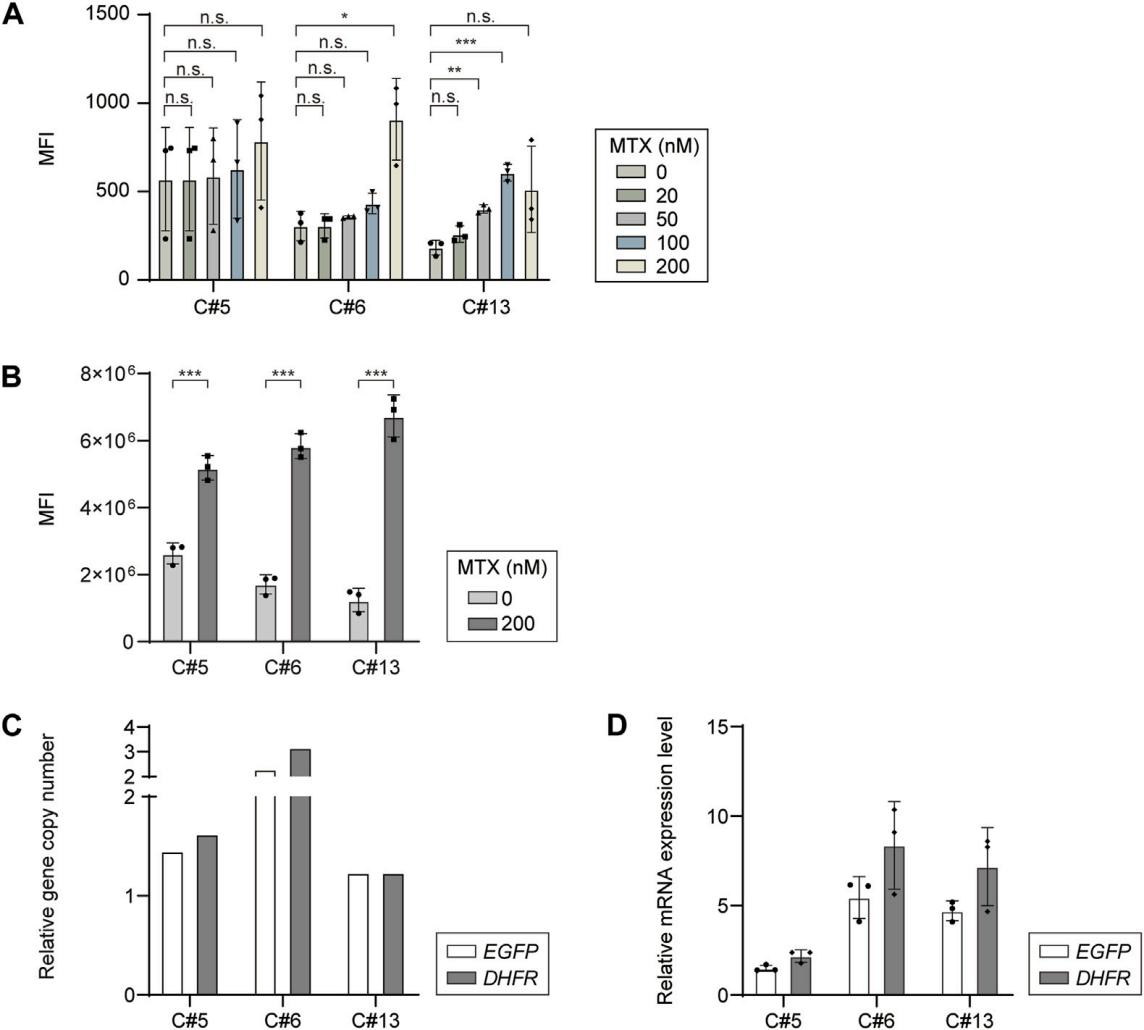
Dihydrofolate reductase/methotrexate (DHFR/MTX) amplification of EGFP in *DHFR* ^-^ recombinase-mediated cassette exchange (RMCE) landing pad master cell line. **(A)** Mean fluorescence intensity (MFI) of EGFP expressing *DHFR* ^−^ RMCE landing pad master cell lines selected at various MTX concentrations. **(B)** MFI profiles of each clone before and after 200 nM MTX selection. Relative **(C)** gene copy number and **(D)** mRNA expression level of *EGFP* and *DHFR*. The gene copy numbers and mRNA levels were calculated by normalization to *vinculin*. The relative values of 200 nM MTX versus 0 nM MTX are shown. The error bars represent standard deviations of three independent experiments. *p ≤ 0.05, **p ≤ 0.01, ***p ≤ 0.001 by an unpaired two-tailed *t*-test; n.s., not significant.

To determine whether DHFR/MTX gene amplification is feasible in targeted integrants with a limited transgene copy number, RMCE landing pad master cell lines expressing EGFP and chDHFR were subjected to a single round of MTX selection for amplification at five different concentrations of MTX (20, 50, 100, 200 nM, and 1 μM). Cells could grow at MTX concentrations up to 200 nM, but not at 1 μM. The EGFP mean fluorescence intensity (MFI) of cell pools selected at various MTX concentrations was measured using flow cytometry to investigate the level of gene amplification (Figure 3A). Among the three clonal landing pad master cell lines, MTX-selected cell pools derived from clones #5 and #6 showed no significant changes in MFI regardless of MTX concentration, except at 200 nM. However, the MFI of clone #13 gradually increased from 0 nM to 100 nM MTX. In the case of 200 nM MTX in clone #13, cell pools initially exhibited decreased MFI compared to those at 100 nM MTX, but a full recovery of cell growth led to a significantly higher MFI than that at the other MTX concentrations (Figure 3B). Interestingly, the basal EGFP expression levels of the three targeted integrants were different to some extent, with approximately two-fold differences between clones #5 and #13, despite the TI of *EGFP* expression cassettes at identical loci (Figure 3B). The difference in the intrinsic expression capacity of the targeted integrants at the *C12orf35* locus may explain the slow recovery of clone #13 at 200 nM MTX. Overall, 200 nM MTX concentration was effective for augmenting EGFP expression, with 1.97-, 3.41-, and 5.42-fold MFI increases in clones #5, #6, and #13, respectively, compared to 0 nM MTX samples, and was thus chosen for further analysis.

To understand the increase in MFI at 200 nM MTX, the relative gene copy numbers and relative mRNA levels of *DHFR* and *EGFP* were analyzed using no-MTX control and 200 nM MTX-selected cell pool samples. The *DHFR* copy numbers of *DHFR*-KO CHO-K1-derived targeted integrants increased by 1.62-, 3.16-, and 1.23-fold at 200 nM MTX compared to those at 0 nM MTX, suggesting that *DHFR* amplification occurred at elevated MTX concentrations (Figure 3C). *EGFP* genes were proportionally coamplified with *DHFR*, increasing by 1.45-, 2.28-, and 1.23-folds in the three targeted integrants (Figure 3C). The relative mRNA levels of *DHFR* and *EGFP* were comparable between the two transgenes, and the trends in fold change were consistent between the relative gene copy numbers and mRNA levels, but at higher relative mRNA levels than the relative gene copy numbers (Figure 3D). Collectively, these data suggest that transgenes can be successfully amplified in *DHFR*-KO CHO-K1-derived targeted integrants using one-step DHFR/MTX gene amplification.

### Characterization of recombinant mAb-producing rCHO cell pools during the DHFR-mediated gene amplification process

**FIGURE 4:**
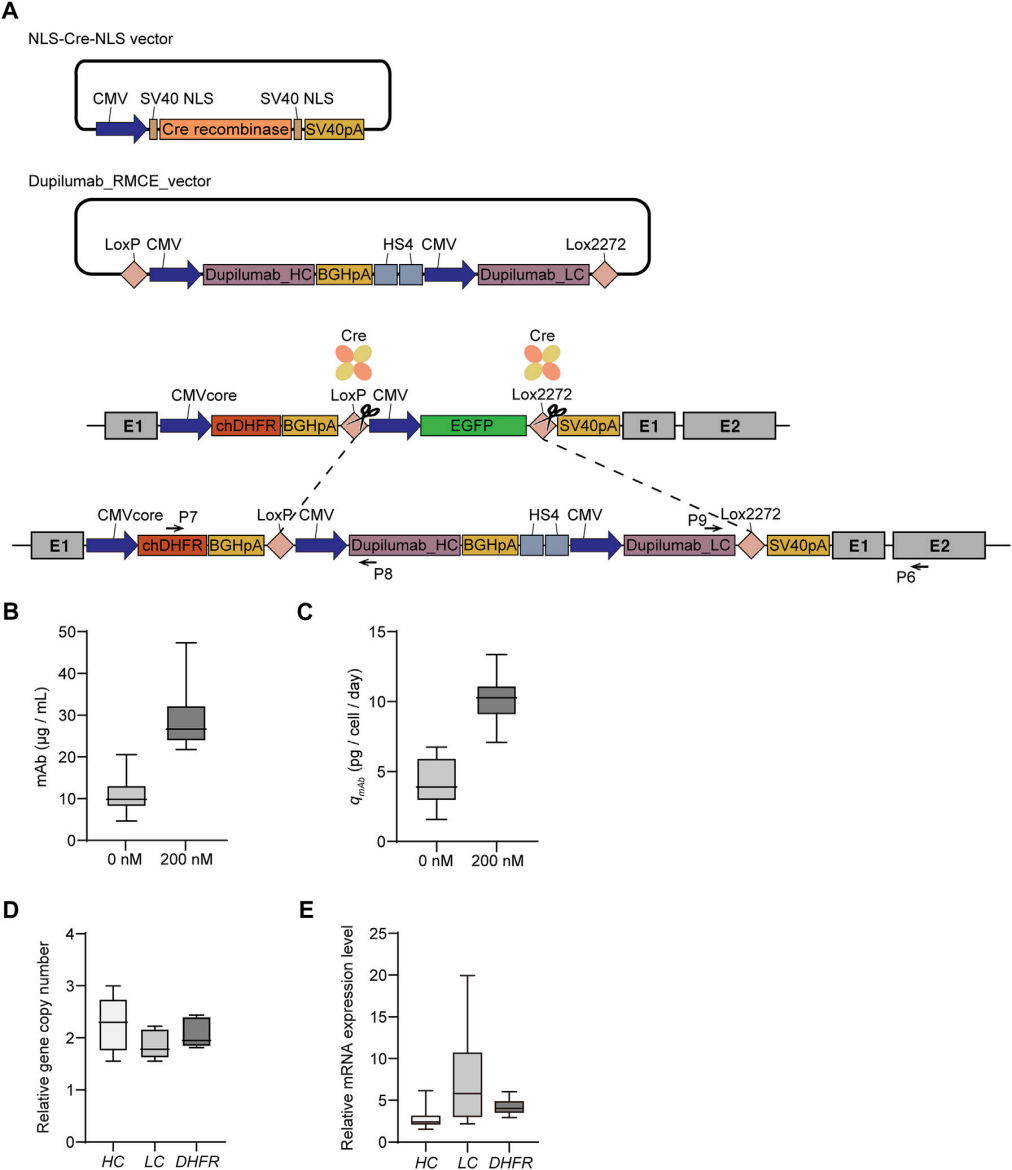
Dihydrofolate reductase/methotrexate (DHFR/MTX) amplification of mAb-producing targeted integrants. **(A)** Schematic diagram for the generation of recombinant cell lines producing mAb by recombinase-mediated cassette exchange (RMCE). Upon cotransfection of Cre recombinase and RMCE donor plasmid containing mAb into *DHFR* ^−^ RMCE landing pad master cell line, EGFP-negative clones were isolated using FACS. Primer positions for 5′/3′-junction PCR to confirm the correct cassette exchange were denoted as black arrows. Profiles of **(B)** mAb production and **(C)** specific mAb productivity (*q*_*mAb*_) of five mAb producing cell lines and their corresponding 200 nM MTX selection pool cells. Relative **(D)** gene copy number and **(E)** mRNA expression level of heavy chain (*HC*), light chain (*LC*), and *DHFR*. The gene copy numbers and mRNA levels were calculated by normalization to *vinculin*. The relative values of 200 nM MTX versus 0 nM MTX are shown. In B and C, the data are from three independent experiments of five clones. In D and E, the data are from five biological replicates.

Based on the feasibility of one-step amplification of intracellular reporter protein expression, we generated recombinant mAb-producing rCHO cell lines to identify DHFR/MTX-mediated amplification of secretory protein. The RMCE landing pad master cell line, clone #6, was cotransfected with the donor plasmid harboring CMV-mAb heavy chain-BGHpA-chicken β-globin insulator HS4-CMV-mAb light chain flanked by LoxP and Lox2272 and Cre recombinase plasmid. EGFP-negative populations were sorted by FACS, followed by limiting dilution (Figure 4A). Cassette exchanges were verified using junction PCR, resulting in the selection of five clones (clones #6, #7, #8, #16, and #17; Supplementary Figure S3). The five selected mAb-producing cell lines were subjected to 200 nM MTX for gene amplification, as described previously.

To assess mAb expression levels in recombinant mAb-producing cell lines with and without MTX selection, five clones and their selection pools were cultured for 4 days in 6-well plates. The mAb concentration and *q*_*mAb*_ of the clones in the absence of MTX were 6.1–14.8 mg/L and 2.3–6.1 pcd, respectively (Figures 4B,C). The 200 nM MTX-selected cell pools showed significantly increased mAb concentration (24.3–37.4 mg/L) and *q*_*mAb*_ (9.1–11.0 pcd), which corresponded to an approximately three-fold increase compared to the 0 nM MTX samples (Figures 4B,C). The gene copy numbers of both *DHFR* and heavy chain (*HC*)/light chain (*LC*) increased with 200 nM MTX selection to a similar extent, in the range of 1.9–2.3 folds, supporting that transgene amplification resulted in enhanced mAb production (Figure 4D). Interestingly, the relative mRNA levels of *DHFR* and *HC*/*LC* were inconsistent with their relative gene copy numbers. The relative mRNA levels of *DHFR* and *HC* in the 200 nM MTX samples were in the range of 3.4–4.8 and 2.2–3.8 folds, respectively, compared to those of 0 nM MTX samples; however, those of LC were in the range of 2.6–14.5 folds, which was highly variable and did not show a meaningful correlation with the relative *LC* copy number of each clone (Figure 4E). To demonstrate that DHFR/MTX-mediated gene amplification could also be applied to non-mAb model proteins, EPO-Fc-producing rCHO cell lines were generated following an identical RMCE landing pad master cell line-mediated CLD process (Supplementary Figure S4). EPO-Fc-producing targeted integrants at the *C12orf35* locus showed *q*_*EPO-Fc*_ of approximately 9 pcd. At 200 nM MTX selection, cells were successfully amplified, with 2.1- and 2.3-fold increases in *DHFR* and *EPO-Fc* copy numbers, respectively, resulting in an average *q*_*EPO-Fc*_ of 18.1 pcd (Supplementary Figure S4). Overall, these data suggest that DHFR/MTX-mediated gene amplification enables the generation of amplified therapeutic protein-producing targeted integrants, leading to augmented target protein production.

### Localization of transgenes in recombinant mAb-producing rCHO cells using FISH

**FIGURE 5:**
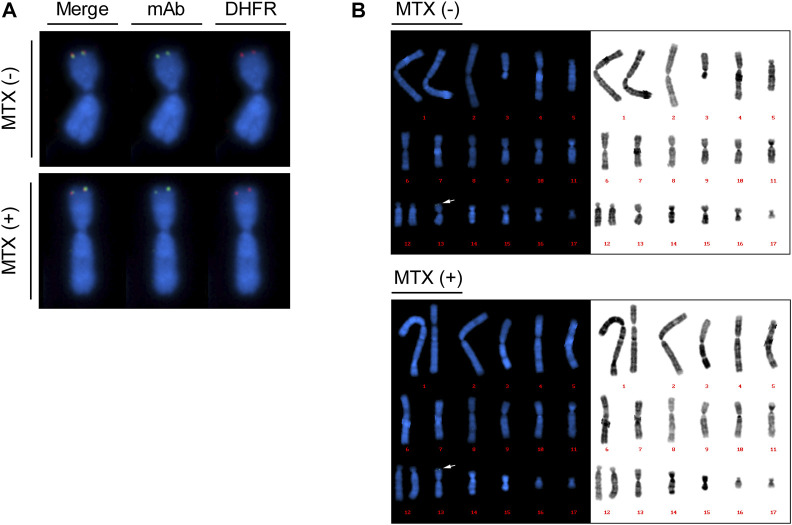
Plasmid localization of mAb-producing recombinant Chinese hamster ovary (rCHO) cells using dual color fluorescence *in situ* hybridization (FISH). Metaphase spreads were prepared for recombinase-mediated cassette exchange (RMCE) landing pad master cell line-derived mAb-producing cell lines with and without MTX selection, followed by dual color FISH. 10 to 20 images were taken for each cell. **(A)** Colocalization of *DHFR* sequences and mAb sequences, identified by red and green signals, respectively. **(B)** Reference karyogram of mAb-producing cell lines. The karyograms are arranged in order of chromosome length. The arrows indicate FISH probe signals shown in **(A)**.

To validate whether the introduced transgenes, including *DHFR* and mAb sequences, were located at identical genomic locations and expected chromosomes before and after MTX amplification, dual-color FISH was performed with probes for *DHFR* and mAb sequences on recombinant mAb-producing cell lines (clone #17) with and without MTX selection. Probe signals from the mAb (green) and DHFR (red) probes were observed at the same position in both the no-MTX control and 200 nM MTX-selected cells (Figure 5A). These signals were located in the telomere region of the short arm of a medium-sized metacentric chromosome (Figure 5B). Karyotype analysis showed that the cells had 19–21 chromosomes, which is consistent with previous studies (Wurm and Hacker, 2011; Vcelar et al., 2018). When the karyograms were arranged in order of chromosome length, most of the cells (90% of no-MTX and 70% of 200 nM MTX-selected cells) contained both FISH signals on the 13th chromosome, and chromosomal abnormalities were found on the chromosome with signals in some cells (Figure 5B and Supplementary Figure S5). In 200 nM MTX-selected cells, we did not observe extended arrays of FISH signals, which are observed in highly amplified rCHO cell lines generated by random integration and step-wise DHFR-mediated gene amplification (Kim and Lee, 1999). These results demonstrate that both *DHFR* and mAb transgenes were integrated at the intended genomic sites, and MTX amplification did not alter the patterns of integrated DNA units.

### Production stability of recombinant mAb-producing rCHO cell pools during long-term cultures in the presence and absence of MTX

**FIGURE 6:**
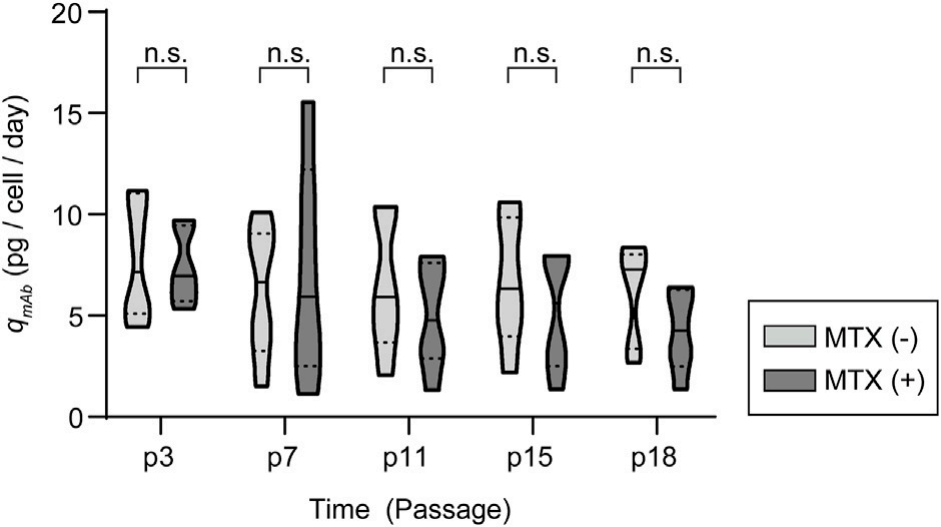
Antibody production characteristics of amplified mAb-producing recombinant Chinese hamster ovary (rCHO) cell pools during long-term cultures in the presence and absence of MTX. Violin plot of *q*_*mAb*_ for five 200 nM MTX-amplified mAb-producing cell pools. n.s., not significant, as determined using two-way ANOVA with p3 for each condition as the control.

To determine the production stability of amplified recombinant mAb-producing rCHO cell pools, five 200 nM MTX selection pools were subcultured for 15 passages, corresponding to 60 days, in 6-well plates in the presence and absence of 200 nM MTX. Overall, *q*_*mAb*_ profiles showed that significant changes in *q*_*mAb*_ did not occur in the presence or absence of MTX during long-term culture, despite fluctuations in values (Figure 6 and Supplementary Figure S6). Compared with *q*_*mAb*_ at the 1^st^ passage (p3), the average *q*_*mAb*_ was in the range of 61–143% and 65–138% in the presence and absence of MTX, respectively. However, among the five 200 nM pools, one mAb-producing cell pool at 200 nM MTX (clone #6) showed a significant decrease in *q*_*mAb*_, retaining only 28% of the initial *q*_*mAb*_ on average after 4^th^ passage (p7). This is in contrast to the absence of a decrease in the other pools (100% of the initial *q*_*mAb*_ on average after 4^th^ passage) despite the presence of MTX.

To evaluate the selection pressure independent loss of mAb productivity in clone #6, the relative gene copy numbers of *DHFR* and *HC*/*LC* were measured using genomic DNA isolated from cell pools at the beginning and end of long-term cultures. Interestingly, clone #6 displayed a remarkable loss of *HC*, retaining only 20% and 9% of initial *HC* gene copies in the presence and absence of MTX, respectively, while the *DHFR* and *LC* copy numbers did not change (data not shown). Meanwhile, the other samples retained most of the gene copies in the presence or absence of MTX. Thus, the antibody production stability of the amplified pools was not related to the presence of MTX, but was correlated with a loss in antibody gene copies during long-term culture.

## Discussion

High-level production of recombinant therapeutic proteins in rCHO cell lines can be achieved through the integration of transgenes into genomic hot spots ensuring high and stable transcriptional levels or amplification of transgenes. In this study, we combined site-specific integration of transgenes into genomic hot spots and the DHFR/MTX gene amplification system to achieve highly producing targeted integrants.

First, we established *DHFR* ^−^ CHO-K1 host cells using CRISPR/Cas9-mediated functional gene KO (Figure 1). Among the widely-used CHO host cell lines, CHO-K1 cells showed a preference for recombinant protein production in terms of cell-specific productivity and product quality, such as galactosylation and sialylation, compared to CHO-S and CHO-DG44 (Reinhart et al., 2019). In addition, compared to the *DHFR* ^−^ host cell lines, CHO-DXB11 and CHO-DG44, CHO-K1 possessed a more active metabolism and increased growth ability (Lakshmanan et al., 2019). Given the various advantageous bioprocessing traits and availability of the RefSeq CHO-K1 genome assembly (Xu et al., 2011), *DHFR* ^−^ CHO-K1 host cells could serve as an alternative *DHFR* ^−^ host cell line to develop high-performance rCHO cell lines using the DHFR-mediated gene amplification system.

Second, based on the *DHFR* ^−^ CHO-K1 host cell, we developed RMCE landing pad master cell lines expressing EGFP and chDHFR by integrating a landing pad into the CHO genomic hot spot, *C12orf35* locus (Figure 2). The *C12orf35* locus was identified from comparative gene expression profiles of high- versus low-rCHO producing cell lines, where stable and high producers had lost the telomeric region of chromosome 8 (Ritter et al., 2016), and targeting the transgene into this locus generated stable cell lines with high productivity and stability (Zhao et al., 2018). In the presence of 200 nM MTX, EGFP expression increased by 3.6 fold, accompanied by 2- and 1.65-fold increases in *DHFR* and *EGFP* copy numbers, respectively (Figure 3).

Third, based on a feasible DHFR-mediated gene amplification system in *DHFR* ^−^ CHO-K1 cells, we established rCHO cell lines that produced recombinant mAbs using RMCE (Figure 4). Targeted integrants at the *C12orf35* locus showed *q*_*mAb*_ of approximately 4.1 pcd in the absence of MTX. Compared to a previous study showing *q*_*mAb*_ of 10–13 pcd targeting the *C12orf35* locus (Zhao et al., 2018), the *q*_*mAb*_ in our study was low, which may be due to different vector configurations and mAb types. Consistent with EGFP expression, *q*_*mAb*_ increased by three folds, together with an approximately two-fold increase in *DHFR* and *mAb* copy numbers, upon 200 nM MTX selection (Figure 4). In addition, most amplified recombinant mAb-producing rCHO cell pools sustained expression levels for 2 months in the presence or absence of 200 nM MTX, which is consistent with the stable EGFP expression in non-amplified RMCE landing pad master cell lines (Supplementary Figure S2) and *C12orf35* targeting mAb producers (Zhao et al., 2018). One unstable mAb-producing cell pool at 200 nM MTX displayed a loss of *HC* genes, which may be related to suboptimal transgene configuration, such as the repetitive use of the CMV promoter and tandemly-arranged *HC*/*LC* expression cassettes.

Insulators are known to increase the stability of transgene expression by blocking the interaction between the promoter and the enhancer or silencer, and have a barrier activity that protects the transgene from the positional effect (Romanova and Noll, 2018). cHS4 is a well-known vertebrate insulator that was used to construct a high-producing rCHO cell line by placing two copies of the 250-bp core of cHS4 between transgene copies (Sergeeva et al., 2020). For the recombinant mAb-producing rCHO cell lines, we inserted two copies of the cHS4 insulator between the *HC* and *LC* expression cassettes to separate each expression cassette, resulting in the configuration of the *DHFR* expression cassette, *HC* expression cassette, cHS4 insulator, and *LC* expression cassette. Upon gene amplification, we observed variable *LC* mRNA expression levels, in contrast to consistent *HC* and *DHFR* expression levels (Figure 4). This may be due to the faster degradation of *HC* mRNA and the relatively faster synthesis of *LC* mRNA (Jiang et al., 2006). However, the effect of the insulator should also be considered, given the relationship between the relative configuration of each expression cassette and mRNA expression levels. The effect of cHS4 is context-dependent (Romanova and Noll, 2018). It only partially protected the CMV promoter from epigenetic silencing in CHO cells and did not increase transgene expression levels in CHO cell culture, compared to its positive effects in other types of mammalian cells and mice (Izumi and Gilbert, 1999; Saunders et al., 2015; Romanova and Noll, 2018). If such a context-dependent effect also occurs at the *C12ort35* locus, the efficiency of the insulator may decrease after amplification, possibly resulting in *LC* transcriptional heterogeneity. A comparative study with and without insulator sequences would further elucidate the effects of insulators and optimal regulatory elements. The use of CHO endogenous insulators, which are derived from the chromosomal location of the exogenous gene-amplified region of the DHFR/MTX-amplified CHO cell line (Takagi et al., 2017), or other promoters that do not alleviate cHS4 barrier activity, could also improve the heterogeneity of *LC* mRNA expression.

The degree of amplification was not as dramatic as that in the MTX-mediated gene-amplified random cell lines. rCHO cell lines producing mAbs, established upon 320 nM MTX selection, showed more than a 50-fold increase in *HC*/*LC* copy numbers (Kim et al., 1998; Kim and Lee, 1999). In the present CRISPR/Cas9-based RMCE landing pad platform cell line, only a several-fold increase in GOI copies was observed. FISH analysis of mAb producers demonstrated that *DHFR* and *mAb* sequences were co-localized at intended chromosomal locations in no-MTX control and 200 nM MTX-selected cells, but did not show clear amplified arrays of FISH signals in 200 nM MTX-selected cells (Figure 5). The discrepancy in the degree of amplification could be due to the different CLD methods and amplification procedures. In contrast to random integration-based CLD, in which a variety of transgenes are integrated at single or multiple genomic sites, site-specific integration-based CLD targets one to two copies of a transgene into predefined genomic sites. In this study, we integrated a single copy of a transgene into a transcriptionally active hot spot locus, followed by gene amplification. High transgene expression from genomic hot spots rendered cells resistant to a low concentration of MTX up to 100 nM without augmentation of transgene expression and up to 200 nM MTX by doubling the transgene copy number (Figures 3, 4). However, the targeted integrants did not grow at 1 μM MTX when cells were exposed to single-step amplification. A limited number of transgene copies may hinder the generation of amplified cells with high copy number. In addition, we used a minimal CMV promoter to express DHFR to allow for more stringent selection conditions. Under selection conditions higher than 200 nM MTX, the relatively low expression level of DHFR resulting from a limited number of amplified *DHFR* copies and low basal promoter activity may not be sufficient to overcome the inhibitory action of MTX. Gene amplification by MTX selection is generally achieved by a gradual increase in MTX concentration, rather than a single round of selection at high levels of MTX (Cacciatore et al., 2010; Noh et al., 2013). To further expand the amplification range, such stepwise gene amplification can be an alternative method, but it is very time-consuming and undermines the advantages of streamlined site-specific integration-based CLD. Therefore, a combination of the current strategy and multicopy TI (Sergeeva et al., 2020) can be a promising strategy that allows one-step selection at high levels of MTX, resulting in high producing targeted integrants harboring a high copy number of the integrated transgene. Furthermore, characterization of the effect of the expression level of DHFR on amplification efficiency together with expression capacity of genomic integration sites can reveal the relationship between the degree of amplification and the initial transgene expression level from specific integration sites without amplification.

In summary, we demonstrated a hybrid CLD platform combining site-specific integration-based CLD and a DHFR-mediated gene amplification system to produce recombinant therapeutic proteins in CHO cells. The use of *DHFR* ^−^ CHO-K1 host cells and single-step MTX amplification enabled accelerated generation of highly producing targeted integrants. Increasing the transgene copy number of established and well-characterized targeted integrants can minimize clonal variation and increase productivity without the need for multicopy targeted integration. The proposed method could provide a new CLD platform for efficient production of biopharmaceuticals, together with further refinement of the amplification range and vector configuration.

## Data Availability

The original contributions presented in the study are included in the article/Supplementary material, further inquiries can be directed to the corresponding author.
