# Supplementary material for: Hybrid cell line development system utilizing site-specific integration and methotrexate-mediated gene amplification in Chinese hamster ovary cells
Source: Front Bioeng Biotechnol. 2022 Sep 15;10:977193. doi: 10.3389/fbioe.2022.977193 (PMC9521551; doi:10.3389/fbioe.2022.977193)
Supplement: Supplementary file 1 [file DataSheet1.docx]

Supplementary Material

# Supplementary Materials and Methods

# Construction of EPO-Fc plasmid

The pcDNA3.1-LoxP-CMV-EPO-Fc-Lox2272 donor was constructed using USER cloning. All primers used for USER cloning are listed in Table S2. pEGFP-C1, coEPO_pcDNA3.1_neo(+) and pcDNA3.4-CMV-Dupilumab_HC were used as the PCR templates for the pcDNA3.1-LoxP-CMV-EPO-Fc-Lox2272 donor. CMV was amplified from pEGFP-C1, EPO from coEPO_pcDNA3.1_neo(+) (Lee et al., 2016), and the Fc region from pcDNA3.4-CMV-Dupilumab_HC.

# Supplementary Figures and Tables

## Supplementary Figures

**
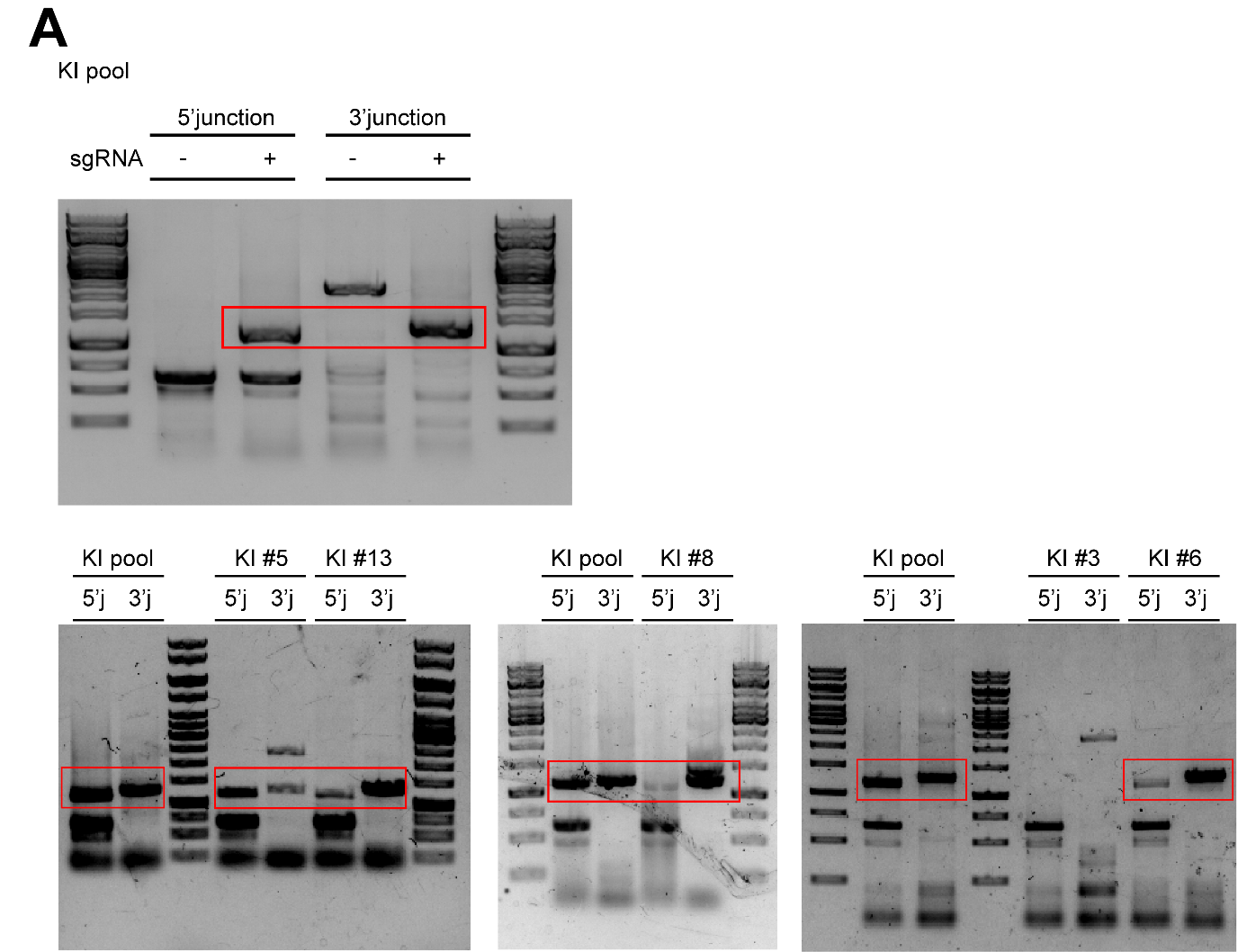
**

**Supplementary Figure 1.** **Validation of the dihydrofolate reductase *(DHFR*)**^-^ **recombinase-mediated cassette exchange (RMCE) landing pad master cell lines by 5′/3′-junction PCR.** 5′/3′-junction PCR analysis of *DHFR*^-^ RMCE landing pad master cell lines. Amplicon sizes of 5′/3′-junction PCR are 1190 bp and 1313 bp, respectively. The knock-in (KI) pool cells were used as positive control when selecting clonal cell lines.

**
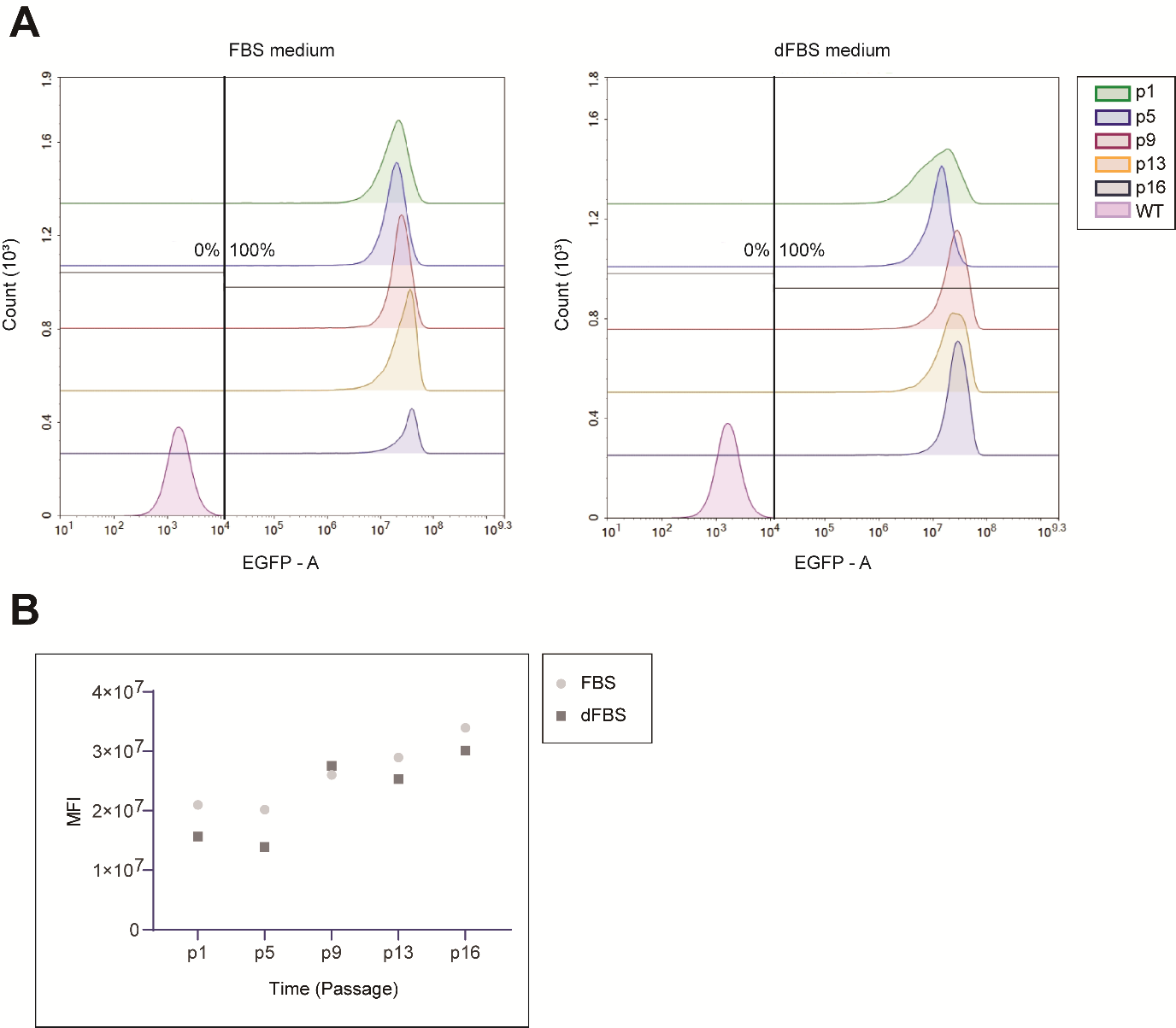
**

**Supplementary Figure 2. Long-term stability of the *EGFP* transgene expression at *C12orf35* locus during long-term cultures in the FBS medium and dFBS medium.** (A) Expression level of EGFP as measured by flow cytometry. Samples (clone #6) were measured every three to four passages. The fluorescence threshold was set to be approximately 0.1% based on the EGFP-negative CHO-K1 wild-type cells. (B) Graphical representation of (A).


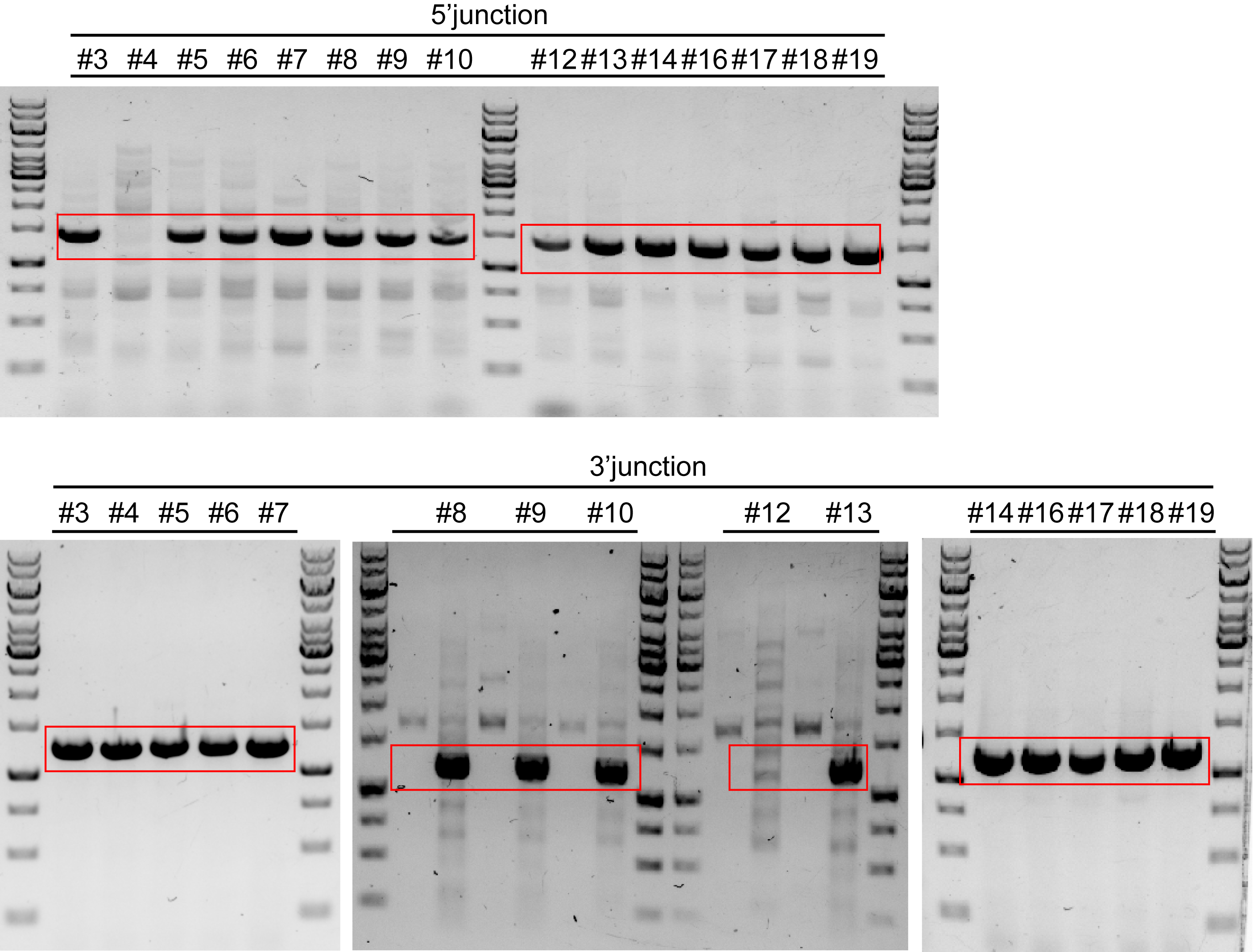


**Supplementary Figure 3.** **Validation of the mAb-producing recombinant Chinese hamster ovary (rCHO) cell line by 5′/3′-junction PCR.** 5′/3′-junction PCR analysis of mAb-producing rCHO cell lines. Amplicon sizes of 5′/3′-junction PCR are 1456 bp and 1250 bp, respectively.

**
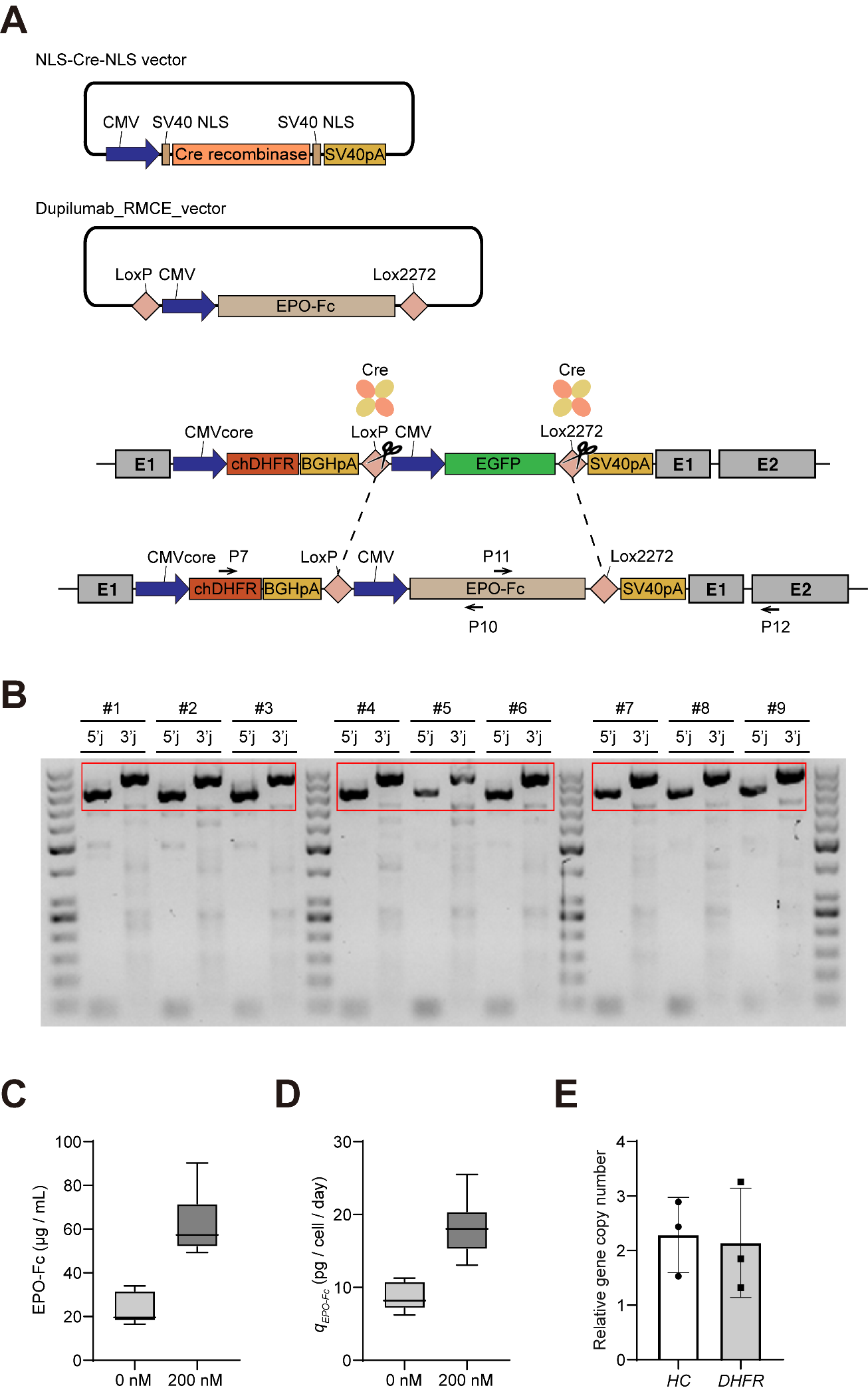
**

**Supplementary Figure 4. Dihydrofolate reductase/methotrexate (DHFR/MTX) amplification of EPO-Fc-producing targeted integrants.** (A) Schematic diagram for the generation of recombinant cell lines producing EPO-Fc by recombinase-mediated cassette exchange (RMCE). Upon cotransfection of Cre recombinase and the RMCE donor plasmid containing EPO-Fc into the DHFR- RMCE landing pad master cell line, EGFP-negative clones were isolated using FACS. Primer positions for 5′/3′-junction PCR to confirm the correct cassette exchange were denoted as black arrows and a simplified name (Supplementary table1). (B) Validation of the cassette exchange by 5′/3′-junction PCR analysis. Amplicon sizes from the 5′/3′-junction PCR are 795 bp and 946 bp, respectively. (C) EPO-Fc production and (D) specific EPO-Fc productivity (*q_EPO-Fc_*) for the three EPO-Fc-producing cell lines and their corresponding 200 nM MTX selection pool cells. (E) Relative gene copy number of the heavy chain (*HC*) and *DHFR*. The gene copy numbers were calculated by normalization to *vinculin*. The relative values of the 200 nM MTX versus 0 nM MTX are shown. The data in C and D is derived from three independent experiments using three clones. The data in E is derived from three biological replicates.

**
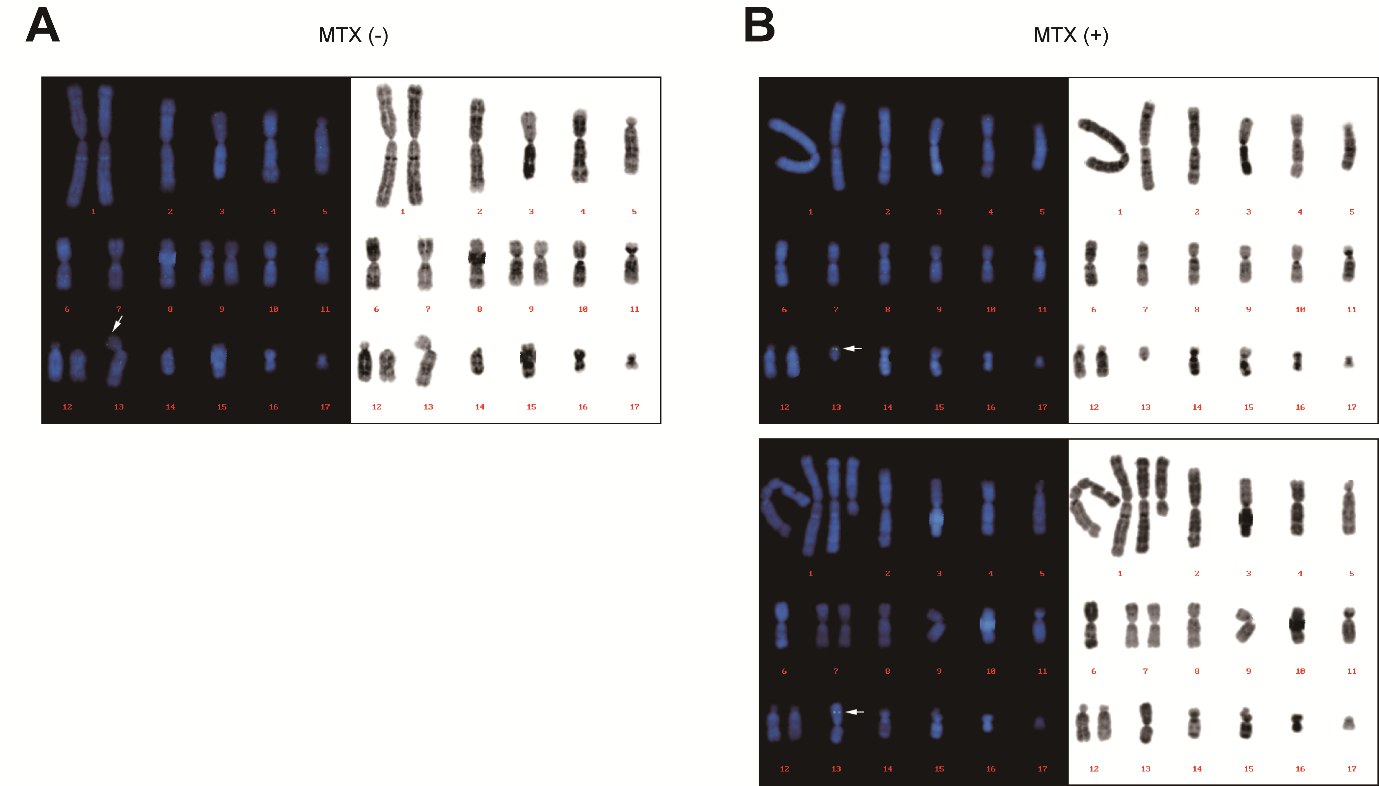
**

**Supplementary Figure 5. Karyogram of mAb-producing cell lines.** Fluorescence in situ hybridization results showed chromosomal abnormalities at the chromosome with signals in some cells. (A) Duplication of the long arm in the absence of MTX. (B) Deletion of the long arm (top) and addition of the short arm (bottom) in the presence of MTX.


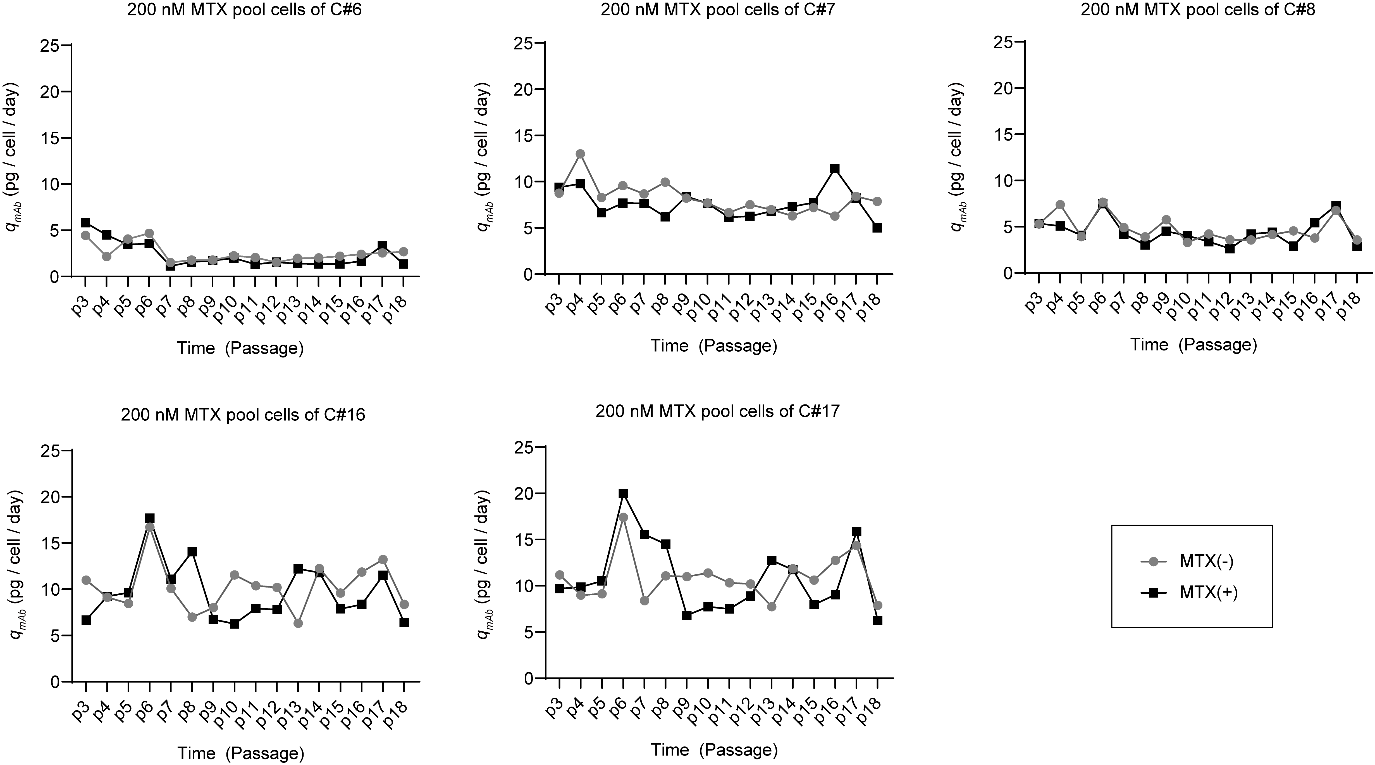


**Supplementary Figure 6. Antibody production characteristics of amplified mAb-producing recombinant Chinese hamster ovary (rCHO) cell pools during long-term cultures in the presence and absence of MTX.**

## Supplementary Tables

**Supplementary Table 1.** Plasmids used in this study

| **T4 ligation** | | | |
| --- | --- | --- | --- |
| **Plasmid name** | **Description** | | **PCR template** |
| 1. sgDHFR-Cas9 | Leading to frame shift mutation and functional gene knockout, Targeting of first exon of CHO-K1 endogenous *DHFR* (sgRNA sequence: fwd- CACCCGTCGCCGTGTCCCAGAATAC rev- AAACGTATTCTGGGACACGGCGACG) | | Chu et al., 2015 |
| 1. sgC12orf-Cas9 | sgRNA expression vector for knock-in into *C12orf35* locus. (sgRNA sequence: fwd- CACCGCCCCCTTACAGCTGTAGATA rev- AAACTATCTACAGCTGTAAGGGGGC) | | Chu et al., 2015 |
| **USER Cloning** | | | |
| **Plasmid name** | **Description** | **DNA Brick information** | **PCR template** |
| 1. pcDNA3.1-CMVcore-chDHFR | Template for Table S1 USER Cloning (C) | CMV core promoter | pEGFP-C1 [Clontech] |
|  |  | chDHFR | CHO-K1 cDNA |
|  |  | BGHpA | pcDNA 3.1(+) [Life Technologies] |
|  |  | Backbone (AmpR, Ori) | pcDNA 3.1(+) [Life Technologies] |
| 1. pcDNA3.1-LoxP-CMV-EGFP-Lox2272 | Template for Table S1 USER Cloning (C) | LoxP_CMV promoter | pEGFP-C1 [Clontech] |
|  |  | EGFP_Lox2272 | pEGFP-C1 [Clontech] |
|  |  | SV40pA | pEGFP-C1 [Clontech] |
|  |  | Backbone (AmpR, Ori) | pcDNA 3.1(+) [Life Technologies] |
| 1. pcDNA3.1-CMVcore-chDHFR-LoxP-CMV-EGFP-Lox2272 | To generate *DHFR*^-^ RMCE landing pad master cell line | C12orf35 homology arm 5’ | CHO-K1 genomic DNA |
|  |  | CMV core promoter | pEGFP-C1 [Clontech] |
|  |  | chDHFR | CHO-K1 complementary DNA |
|  |  | BGHpA | pcDNA 3.1(+) [Life Technologies] |
|  |  | LoxP_CMV promoter | pEGFP-C1 [Clontech] |
|  |  | EGFP_Lox2272 | pEGFP-C1 [Clontech] |
|  |  | SV40pA | pEGFP-C1 [Clontech] |
|  |  | Backbone (AmpR, Ori) | pcDNA 3.1(+) [Life Technologies] |
| 1. pcDNA3.1- LoxP-CMV- Dupilumab_HC-BGHpA- cHS4-CMV- Dupilumab_LC-Lox2272 | To generate mAb-producing rCHO cell | LoxP_CMV promoter | pEGFP-C1 [Clontech] |
|  |  | Dupilumab_HC | Kim et al., 2019 |
|  |  | BGH pA_HS4 | Addgene #154834 Sergeeva et al., 2020 |
|  |  | HS4 | Addgene #154834 Sergeeva et al., 2020 |
|  |  | CMV promoter | pEGFP-C1 [Clontech] |
|  |  | Dupilumab_LC | Kim et al., 2019 |
|  |  | Backbone (AmpR, Ori) | pcDNA 3.1(+) [Life Technologies] |
| 1. pcDNA3.1-LoxP-CMV-EPO-Fc-Lox2272 | To generate EPO-Fc-producing rCHO cell | CMV promoter | pEGFP-C1 [Clontech] |
|  |  | EPO | coEPO pcDNA3.1 neo(+) (Lee et al., 2016) |
|  |  | Fc region | Dupilumab_HC |
|  |  | Backbone (AmpR, Ori) | pcDNA 3.1(+) [Life Technologies] |

**Supplementary Table 2.** Primer sequences

| **Primer name** | **Simplified** | | **Purpose** | **Sequence (5'-3')** |
| --- | --- | --- | --- | --- |
| **Donor plasmid** | | | | |
| pcDNA3.1 backbone_O5_fwd | - | | For all donor plasmid in this study | ACTTGCGUACTTTTCGGGGAAATGTGCG |
| pcDNA3.1 backbone_LA_rev | - | | For all donor plasmid in this study | ACACCGACUCAGTCGGGAAACCTGTCGTG |
| CMV core_LA_fwd | - | | Table S1 USER cloning (A) | AGTCGGTGUCGCCCCATTGACGCAAATG |
| CMV_LB_rev | - | | Table S1 USER cloning (A), (D), (E) | ACGCTGCTUGATCTGACGGTTCACTAAACCA |
| chDHFR_LB_fwd | - | | Table S1 USER cloning (A) | AAGCAGCGUACCATGGTTCGACCGCTGAACTGC |
| chDHFR_LC_rev | - | | Table S1 USER cloning (A) | ATGACGTCUTTAGCCTTTCTTCTCATAGAC |
| BGHpA_LC_fwd | - | | Table S1 USER cloning (A) | AGACGTCAUCTGTGCCTTCTAGTTGCCA |
| BGHpA_O5_rev | - | | Table S1 USER cloning (A) | ACGCAAGUCCATAGAGCCCACCGCATC |
| Loxp-CMV_LA_fwd | - | | Table S1 USER cloning (B), (C), (D), (E) | AGTCGGTGUATAACTTCGTATAGCATACATTATACGAAGTTATATAGTAATCAAT |
| Lox2272-EGFP_LC_rev | - | | Table S1 USER cloning (B) | ATGACGTCUATAACTTCGTATAAAGTATCCTATACGAAGTTATTTATCTAGATCCGGTGGATC |
| SV40pA_LC_fwd | - | | Table S1 USER cloning (B) | AGACGTCAUTGATCATAATCAGCCATACC |
| SV40pA_O5_rev | - | | Table S1 USER cloning (B) | ACGCAAGUTAAGATACATTGATGAGTTTG |
| C12orf35 5' arm_LA_fwd | - | | Table S1 USER cloning (B) | AGTCGGTGUTTTCAGCTGTCTCCCACA |
| C12orf35 5' arm_LB_rev | - | | Table S1 USER cloning (C) | ACGCTGCTUCTACAGCTGTAAGGGGG |
| CMV_core_LB_fwd | - | | Table S1 USER cloning (C) | AAGCAGCGUCGCCCCATTGACGCAAATG |
| BGH pA_LE_rev | - | | Table S1 USER cloning (C) | AAAGCCTAUCCATAGAGCCCACCGCATCC |
| LoxP_LE_fwd | - | | Table S1 USER cloning (C) | ATAGGCTTUATAACTTCGTATAGCATACA |
| SV40pA_LC_rev | - | | Table S1 USER cloning (C) | ATGACGTCUTAAGATACATTGATGAGTTTG |
| C12orf35 3' arm_LC_fwd | - | | Table S1 USER cloning (C) | AGACGTCAUATATGGAAGCCAACATGTGC |
| C12orf35 3' arm_O5_rev | - | | Table S1 USER cloning (C) | ACGCAAGUCCAACAGCTACATCTGAAGA |
| LB_Kozak_Dupilumab_HC_fwd | - | | Table S1 USER cloning (D) | AAGCAGCGUCGCCACCATGGGATGGAGCT |
| Dupilumab_HC_O3_rev | - | | Table S1 USER cloning (D) | AGCGCTGGUCTATTTACCCGGGGACAGGG |
| BGHpA_O3_fwd | - | | Table S1 USER cloning (D) | ACCAGCGCUCTGTGCCTTCTAGTTGCCAGCCA |
| HS4_O2_rev | - | | Table S1 USER cloning (D) | ATCGCACUCGTGCACTCAGCCTAAAGCT |
| O2_HS4_fwd | - | | Table S1 USER cloning (D) | AGTGCGAUATTTAAATGGGGACAGCCCC |
| HS4_LF_rev | - | | Table S1 USER cloning (D) | ATCCACGTUCGTGCACTCAGCCTAAAGCT |
| LF_CMV_fwd | - | | Table S1 USER cloning (D) | AACGTGGAUATAGTAATCAATTACGGGGT |
| CMV_LC_rev | - | | Table S1 USER cloning (D) | ATGACGTCUGATCTGACGGTTCACTAAACCA |
| LC_Kozak_Dupilumab_LC_fwd | - | | Table S1 USER cloning (D) | AGACGTCAUCGCCACCATGGAGACAGACA |
| Dupilumab_LC_Lox_2272_O5_rev | - | | Table S1 USER cloning (D) | ACGCAAGUATAACTTCGTATAAAGTATCCTATACGAAGTTATCTAACACTCTCCCCTGTTGA |
| EPO_fwd | - | | Table S1 USER cloning (E) | ATGGGAGTGCACGAGTGTCCTGC |
| EPO_overlap_rev | - | | Table S1 USER cloning (E) | ATTTGGGCTCTCTATCGCCGGTCCGGCAAG |
| Fc_overlap_fwd | - | | Table S1 USER cloning (E) | CGGCGATAGAGAGCCCAAATCTTGTGACAA |
| Fc_rev | - | | Table S1 USER cloning (E) | CTATTTACCCGGGGACAGGG |
| Kozak_EPO_LB_fwd | - | | Table S1 USER cloning (E) | AAGCAGCGUCGCCACCATGGGAGTGCACGAGTGTCC |
| Dupilumab_HC_Lox_2272_O5_rev | - | | Table S1 USER cloning (E) | ACGCAAGUATAACTTCGTATAAAGTATCCTATACGAAGTTATCTATTTACCCGGGGACAGGG |
| **DHFR KO validation** | | | | |
| CHO-K1(WT)_Dhfr_k/o_seq_primer(fwd) | P1 | DHFR KO validation | | GTGGCCTCCGATTCACAAGT |
| K1(WT)_Dhfr_k/o_seq_primer(rev) | P2 | DHFR KO validation | | ACTGAGGAGGTGGTGGTCAT |
| **5’/3’ junction PCR** | | | | |
| C12orf35 TI_5'arm_Junction fwd | P3 | | Amplicon for 5’ junction PCR of Landing pad | CCCCAAACCCACCATATTCT |
| C12orf35 TI_5'arm_Junction rev | P4 | | Amplicon for 5’ junction PCR of Landing pad | TCCTTGTGGTGGTTCCTTGAG |
| C12orf35 TI_3'arm_Junction fwd | P5 | | Amplicon for 3’ junction PCR of Landing pad | CCCGACAACCACTACCTGAG |
| C12orf35_TI_3'Junction_rev | P6 | | Amplicon for 3’ junction PCR of Dupilumab_LC and Landing pad | CCACAGCAATCTGAAGCTCT |
| chDHFR_c12orf_5junction_fwd | P7 | | Amplicon for 5’ junction PCR of Dupilumab_HC, EPO-FC | CCCAGAATATGGGCATCGGC |
| Dupilumab_c12orf_5junction_rev | P8 | | Amplicon for 5’ junction PCR of Dupilumab_HC | TGTACACCTGTAGCTGTTGC |
| CL_seq_primer_fwd | P9 | | Amplicon for 3’ junction PCR of Dupilumab_LC | GACAGCAAGGACAGCACCTA |
| EPO_overlap_rev | P10 | | Amplicon for 5’ junction PCR of EPO-Fc | CGGCGATAGAGAGCCCAAATCTTGTGACAA |
| EPO_Fc 3'Junction fwd | P11 | | Amplicon for 3’ junction PCR of EPO-Fc | TTCAACTGGTACGTGGACGG |
| EPO_Fc 3'Junction rev | P12 | | Amplicon for 3’ junction PCR of EPO-Fc | TACTAGTGCTCTGCCACTGC |
| **Quantitative real-time PCR** | | | | |
| chDHFR_qPCR_F | - | | Amplicon for exon region of chDHFR | GAATATGGGCATCGGCAAGA |
| chDHFR_qPCR_R | - | | Amplicon for exon region of chDHFR | GGTTCTGTTTACCTTCCACTGA |
| EGFP qPCR fwd | - | | Amplicon for exon region of EGFP | GAACCGCATCGAGCTGAA |
| EGFP qPCR rev | - | | Amplicon for exon region of EGFP | TGCTTGTCGGCCATGATATAG |
| Vinculin_1F | - | | Amplicon for exon region of Vinculin1 | GCTGGTTGCTAAGAGGGAGG |
| Vinculin_1R | - | | Amplicon for exon region of Vinculin1 | ATCAGAGGCAGCTTTCACGG |
| mAB_HC_qPCR_fwd | - | | Amplicon for exon region of CH3 of Dupilumab and EPO-Fc | CAGCCGGAGAACAACTACAA |
| mAB_HC_qPCR_rev | - | | Amplicon for exon region of CH3 of Dupilumab and EPO-Fc | CATCACGGAGCATGAGAAGA |
| mAB_LC_qPCR_fwd | - | | Amplicon for exon region of CL of Dupilumab | GTTGTGTGCCTGCTGAATAAC |
| mAB_LC_qPCR_rev | - | | Amplicon for exon region of CL of Dupilumab | TCCTGCTCTGTGACACTCT |
| **Sanger sequencing** | | | | |
|  | | | **Donor plasmid** |  |
| CMV core_LA_fwd | - | | Table S1 (B) | AGTCGGTGUCGCCCCATTGACGCAAATG |
| BGHpA_O5_rev | - | | Table S1 (B) | ACGCAAGUCCATAGAGCCCACCGCATC |
| LoxP-CMV_LA_fwd | - | | Table S1 (C), (F) | AGTCGGTGUATAACTTCGTATAGCATACATTATACGAAGTTATATAGTAATCAATTACGGGGT |
| SV40pA_O5_rev | - | | Table S1 (C) | ACGCAAGUTAAGATACATTGATGAGTTTG |
| 5arm_seq_1_fwd | - | | Table S1 (D), (E), (F) | CCTGATTCTGTGGATAACCG |
| CMV_core_LB_fwd | - | | Table S1 (D) | AAGCAGCGUCGCCCCATTGACGCAAATG |
| LoxP_LE_fwd | - | | Table S1 (D) | ATAGGCTTUATAACTTCGTATAGCATACA |
| C12orf35 3' arm_LC_fwd | - | | Table S1 (D) | AGACGTCAUATATGGAAGCCAACATGTGC |
| 3arm_seq_1_rev | - | | Table S1 (D), (E), (F) | CCCAACTGATCTTCAGCATC |
| LB_Kozak_Dupilumab_HC_fwd | - | | Table S1 (E) | AAGCAGCGTCGCCACCATGGGATGGAGCT |
| Dupilumab_HC_seq_rev | - | | Table S1 (E) | ATGGCATAGTCTCTAAAGGT |
| CH1_seq_primer_fwd | - | | Table S1 (E) | CAGCAGCGTGGTGACCGTGC |
| CH3_seq_primer_fwd | - | | Table S1 (E) | GTCTTCTCATGCTCCGTGAT |
| Dupilumab_LC_seq_rev | - | | Table S1 (E) | GGAGACTGGGTCATCACGAT |
| LC_Kozak_Dupilumab_LC_fwd | - | | Table S1 (E) | AGACGTCATCGCCACCATGGAGACAGACA |
| Kozak_EPO_LB_fwd | - | | Table S1 (F) | AAGCAGCGUCGCCACCATGGGAGTGCACGAGTGTCC |
| Fc_overlap_fwd | - | | Table S1 (F) | CGGCGATAGAGAGCCCAAATCTTGTGACAA |
| Dupilumab_HC_Lox_2272_O5_rev | - | | Table S1 (F) | ACGCAAGUATAACTTCGTATAAAGTATCCTATACGAAGTTATCTATTTACCCGGGGACAGGG |
| EPO_overlap_rev | - | | Table S1 (F) | ATTTGGGCTCTCTATCGCCGGTCCGGCAAG |
| CMV_LB_rev | - | | Table S1 (F) | ACGCAAUTATAACTTCGTATAAAGTATCCTATACGAAGTTATCCATAGAGCCCACCGCATCC |

**Supplementary Table 3**. *DHFR* genomic sequence information in knockout clones

| **Clones** | ***DHFR* sequence (NW_003614442)** | **Indel** | **Frequency** |
| --- | --- | --- | --- |
| **WT** | ACTGCATCGTCGCCGTGTCCCAGAATATGGGCATCGGCAAGAACGGAGACCTTCCCTGGCCAATGCTCAGG | - | - |
| **KO#4** | ACTGCATCGTCGCCGTG**----------------------------------------**AGAACGGAGACCTTCCCTGGCCAATGCTCAGG | -22 bp | 8/8 |
| **KO#13** | ACTGCATCGTCGCCGTG**----------------------------------------**AGAACGGAGACCTTCCCTGGCCAATGCTCAGG | -22 bp | 1/7 |
|  | ACTGCATCGTCGCCGTGTCCCAGAAT**--------------------------------------------------------------------**TGCTCAGG | -37 bp | 1/7 |
|  | ACTGCATCGTCGCCGTGTCCCAGT**------------------------------------------------------------------------**TGCTCAGG | -39 bp | 5/7 |
| **KO#14** | ACT**-------------------------------------------------**GCATCGGCAAGAACGGAGACCTTCCCTGGCCAATGCTCAGG | -27 bp | 2/2 |
| **KO#16** | ACT**-------------------------------------------------**GCATCGGCAAGAACGGAGACCTTCCCTGGCCAATGCTCAGG | -27 bp | 5/5 |

# REFERENCES

Chu, V. T., Weber, T., Wefers, B., Wurst, W., Sander, S., Rajewsky, K., and Kühn, R. (2015). Increasing the efficiency of homology-directed repair for CRISPR-Cas9-induced precise gene editing in mammalian cells. *Nat. Biotechnol.* 33, 543-548.

Kim, J. E., Jung, K., Kim, J. A., Kim, S. H., Park, H. S., and Kim, Y. S. (2019). Engineering of anti-human interleukin-4 receptor alpha antibodies with potent antagonistic activity. *Sci. Rep.* 9, 1-12.

Lee, J. S., Grav, L. M., Pedersen, L. E., Lee, G. M., and Kildegaard, H. F. (2016). Accelerated homology-directed targeted integration of transgenes in Chinese hamster ovary cells via CRISPR/Cas9 and fluorescent enrichment. *Biotechnol. Bioeng.* 113, 2518-2523.

Sergeeva, D., Lee, G. M., Nielsen, L. K., and Grav, L. M. (2020). Multicopy targeted integration for accelerated development of high-producing Chinese hamster ovary cells. *ACS Synth. Biol.* 9, 2546-2561.
